# Functional Variants Associated With *CMPK2* and in *ASB16* Influence Bovine Digital Dermatitis

**DOI:** 10.3389/fgene.2022.859595

**Published:** 2022-06-27

**Authors:** Diana Oelschlaegel, Monika Wensch-Dorendorf, Grit Kopke, Roswitha Jungnickel, Benno Waurich, Frank Rosner, Dörte Döpfer, Bertram Brenig, Hermann H. Swalve

**Affiliations:** ^1^ Group Animal Breeding, Institute of Agricultural and Nutritional Sciences, Martin-Luther-University Halle-Wittenberg, Halle, Germany; ^2^ Department of Medical Sciences, School of Veterinary Medicine, University of Wisconsin, Madison, WI, United States; ^3^ Institute of Veterinary Medicine, Georg-August-University Göttingen, Göttingen, Germany

**Keywords:** bovine digital dermatitis, M-stage system, GWAS, CMPK2, ASB16

## Abstract

Bovine digital dermatitis (BDD) is an infectious disease of the hoof in cattle with multifactorial etiology and a polygenic influence on susceptibility. With our study, we identified genomic regions with the impact on occurrence and development of BDD. We used 5,040 genotyped animals with phenotype information based on the M-stage system for genome-wide association. Significant associations for single-nucleotide polymorphisms were found near genes *CMPK2* (chromosome 11) and *ASB16* (chromosome 19) both being implicated in immunological processes. A sequence analysis of the chromosomal regions revealed rs208894039 and rs109521151 polymorphisms as having significant influence on susceptibility to the disease. Specific genotypes were significantly more likely to be affected by BDD and developed chronic lesions. Our study provides an insight into the genomic background for a genetic predisposition related to the pathogenesis of BDD. Results might be implemented in cattle-breeding programs and could pave the way for the establishment of a BDD prescreening test.

## Introduction

Bovine digital dermatitis (BDD), first described over 40 years ago (Cheli and Mortellaro, 1974), is an infectious disease of the bovine foot, frequently occurring in the plantar skin bordering the interdigital cleft of the rear leg (Holzhauer et al., 2008). The disease has become one of the most common claw diseases associated with severe lameness in cattle. BDD is difficult to manage as it is persistent, has different clinical manifestations, and high recurrence after therapy (Berry et al., 2012). Even though the pathogenesis of BDD has not been completely determined yet, a multifactorial etiology and influence of several microbes with a primary impact of *Treponema* spp. is highly probable (Evans et al., 2014; Zinicola et al., 2015).

The disease has been reported as a worldwide problem in dairy cows and beef cattle (Orsel et al., 2018). In addition to economic effects for the affected herds, a negative impact on performance and well-being of cattle has been described. New strategies for prevention of BDD are needed, given that no efficient vaccines exist to date (Evans et al., 2014), and the application of (topical) antibiotics and chemicals in footbaths induces bacterial resistances, pollutes the environment, and negatively impacts the health of the operator (Laven and Logue, 2006; Speijers et al., 2010). Systems for standardized recording of disease stages have been developed to describe visual changes and pain in the skin as well as the response to treatment (Döpfer et al., 1997; Shearer and Hernandez, 2000). The use of the well-established M-scoring system (Döpfer et al., 1997) can be applied to record frequency and progress of affected animals for an assessment of risk factors influencing BDD in a herd as well as for genetic evaluations (Schöpke et al., 2015; Tremblay et al., 2016). Moderate-to-high heritability estimates and a varying immune response for different cow types regarding their susceptibility to BDD support the influence of host genetic factors (Scholey et al., 2012; Gomez et al., 2014). Only a limited number of genome-wide association studies or gene expression studies have been undertaken for the case of BDD (van der Spek et al., 2015; Biemans et al., 2019; Croué et al., 2019; Lai et al., 2020). Most of these studies lack in the size of the experiment or field data necessary to identify chromosomal regions by means of GWAS (genome-wide association study) and/or fail to use repeated observations indispensable to account for the complex etiology of BDD. Hence, the results were mostly heterogeneous, and findings of one study could hardly be validated by other studies (Biemans et al., 2019; Croué et al., 2019; Sánchez-Molano et al., 2019). Currently, some candidate genes implicated in inflammatory processes have been described (Tuschil et al., 1992; Refaai et al., 2013; El-Shafaey et al., 2017). Furthermore, gene expression analysis of BDD lesions revealed an increased expression of some cytokines, as well as a reduced expression of keratins, and keratin-associated genes (Scholey et al., 2013). These findings imply a mismanaged local immune response to the bacterial infection and aberrant migration and proliferation behavior of keratinocytes. However, causal mutations associated with the occurrence of BDD have not been described yet for any of these genes. The objective of this study was to identify chromosomal regions and candidate genes with causal variants influencing BDD based on genotyping of single-nucleotide polymorphisms (SNP). We used distinct disease traits defined using the M-stages for BDD stages (Döpfer et al., 1997; Berry et al., 2012) and a total of 5,040 genotyped animals with phenotype information for a genome-wide association study. Looking forward, our knowledge about SNP association could pave the way for the establishment of a much needed BDD prescreening test.

## Materials and Methods

### Animals and Ethical Statement

All cows considered for this study belonged to the Holstein breed, the worldwide dominating dairy breed, and were from commercial herds in northeastern Germany. Phenotypes were collected as scores based on visual inspection and thus completely non-invasive. Cows were not specifically restrained while scoring but rather the possible occurrence of BDD lesions was scored while cows were milked, according to the usual daily routine of a commercial dairy farm. This daily routine was completely unchanged for the scoring and not altered in any way, for example, timewise or by the order of animals for scoring. SNP array genotypes were available for all cows included in the study and had been based on blood samples taken for projects of the respective herdbook association. Thus, the results of SNP array genotyping were available from a central database. Likewise, DNA samples that had been stored as retention samples after SNP array genotyping were available from the two laboratories that had carried out the genotyping with the consent of the two herdbook associations involved, namely, RinderAllianz GmbH and RBB Rinderproduktion Berlin-Brandenburg GmbH. The collection of samples was approved by the Lower Saxony State Office for Consumer Protection and Food Safety (33.19-42502-05-17A196) according to §8a Abs. 1 Nr. 2 of the German Animal Protection Law.

### Study Design and Data Management

Scoring of phenotypes was conducted in two batches. The first batch consisted of seven large dairy herds visited between October 2015 and April 2016. Herds were selected based on the type of the milking parlor. External rotary parlors were preferred to enable BDD assessment from the outside of the milking carousel. Six of the herds had external rotary parlors, while one farm was equipped with a side-by-side milking parlor. Herds were selected for phenotyping from a pool of 56 herds participating in a scientific project covering a broad range of phenotypic traits for examinations of feasibility of genomic selection based on individual cow phenotypes. In these herds, 20,000 cows, focusing on young cows in their first lactation, had been genotyped based on a protocol consisting of random sampling. Cows were drawn to be included in the study before phenotyping started.

The second batch of data consisted of cows in a further set of six large dairy farms visited between April 2018 and July 2018. Participating herds in this case had enrolled in a genotyping program organized by their herdbook association with the goal of obtaining genomic breeding values for their livestock. The genotyping program was organized in such a way that young stock and cows during first lactation were genotyped by birth year cohorts without any exemptions within birth year. Thus, no selection was practiced. Depending on the time when the herd had enrolled, genotypes were available at least for first lactation and partly also for cows in second lactation.

Every farm in both batches of data was visited three times by a team of three trained persons at intervals of 3 weeks. The team leader and third author is a veterinarian specifically trained for assessing BDD status and was present during all farm visits. All cows present at the time of milking were examined in each herd. As cows in a commercial dairy herd may enter or leave the herd based on herd management, not all cows could be assessed during all three visits. The study group included only cows that were inspected at least twice, with a clear majority of 81.7% being scored three times. After phenotype collection, the resulting data were merged with the genotypes available thus forming the data set useable for analysis. By mere coincidence, after merging phenotypes with genotypes, the first and the second batch of data resulted in 2,520 cows each.

Every cow received a score for the M-stage (M0, M2, or M4) (Döpfer et al., 1997) and a score for signs of chronicity, be it hyperkeratotic or proliferative. A detailed illustration of the scoring system with corresponding symptoms is shown in Supplementary Figure S1. Phenotypes used in our study were defined as follows: for trait TBIN, individuals were coded as 0 if all repetitive scores were M0 and coded as 1 otherwise. For trait TBINA, code 0 was defined as mentioned earlier while a code of 1 denoted an M2 case in any of the repetitive scores. Trait TCHRONA was defined as code = 0 if no chronic proliferative signs were detected in any of the repeated scores and coded = 1 otherwise.

DNA extraction and genotyping was carried out by the IFN Schönow e.V (Institut für Fortpflanzung landwirtschaftlicher Nutztiere Schönow e.V., Bernau, Germany) and the Zentrum für molekulare Diagnostik ZMD at the Institute of Veterinary Medicine (Georg-August-University Göttingen, Germany). The majority of all cows in batches 1 and 2 were genotyped using the Illumina EuroG10K BeadChip versions 4 and 5 (Illumina Inc., San Diego, CA, United States). A subset of 516 animals within batch 1 was analyzed using the Illumina BovineSNP50 BeadChip versions 1 and 2. Quality control was set as call rate >90% and minor allele frequencies >1%. Imputation from the 10-K SNP chip information to the 50-K variant *via* the bioinformatics tool FImpute version 2.2 (Sargolzaei et al., 2014) with an accuracy of 99.5% was performed by Vereinigte Informationssysteme Tierhaltung (VIT, Verden/Aller, Germany). Finally, a total of 45,613 SNP markers could be employed for a genome-wide association approach.

### Genome-Wide Association Study

For GWAS, the software based on the BLUPF90 software family (Misztal et al., 2002) adapted for genomic analyses (Aguilar et al., 2011) was employed in the form of GBLUP analysis. Modeling was performed according to a threshold model as applicable for binary traits. THRGIBBSF90 was used to estimate the variance components and then to predict the GEBV. The SNP effects were calculated using postGSf90 software. The single-trait threshold animal model for GBLUP/GWAS is as follows:

$$PR\left(Y_{ijkl}=1\right)=\varphi \left(\mu +herd_{i}+parity_{j}+DIM_{k}+animal_{l}\right),$$ (1)

where *PR* is the probability of occurrence, *y*_*ijkl*_ is the vector of phenotypic observations coded as binary traits across 2 or 3 observations, *φ* is the probit link function, μ is the overall mean, *herd*_*i*_ is the fixed effect of the farm (*i* = 1, …, 7 for batch 1 and *i* = 1, …, 13 for batch 1 + 2 combined), *parity*_*j*_ is the fixed effect for parity (primipar or multipar), *DIM*_*k*_ is the fixed effect for lactation stage grouped into classes (*k* = 1, …, 8 for DIM <50, 50–99, 100–149, 150–199, 200–249, 250–299, 300–349, and >350), and *animal*_*l*_ is the random additive genetic effect of animals (*l* =1, …,5040). For random additive genetic effects, the covariance structure is given by the matrix G. G was set up comprising all cows with phenotypes and genotypes and their genotyped sires as available from the central database. To control for multiple testing, the false discovery rate (FDR) was used (Benjamini and Hochberg 1995). An initial GWAS analysis was conducted after sampling of batch 1 had been completed, and a final analysis included all individuals from batch 1 and 2. As a main result from GWAS, two SNPs with significant effects were obtained for chromosomes 11 and 19 after analysis of batch 1. For identification of animals to be sequenced for candidate genes, the two positions of these SNPs subsequently were subject to an analysis of phased haplotypes. The procedure of the haplotype analysis along with detailed results is presented in detail in Supplementary Tables S1, S2. In brief, phased haplotype data inferred from genotype information of the study animals along with their sires were provided by the Vereinigte Informationssysteme Tierhaltung (VIT, Verden/Aller, Germany), having employed the software FImpute version 2.2 (Sargolzaei et al., 2014). For all 2,520 genotyped cows in batch 1 and both regions, a window of 10 SNPs surrounding the target SNP, (HapMap60356-rs29024194, BTA11, and BTA-45551-no-rs, BTA19) was selected, and haplotypes of all animals were determined.

In a first run of haplotype analysis, all individuals homozygous for a given haplotype formed one effect class while the vast majority of animals were assigned to a class “heterozygous” as they showed some form of heterozygosity for the haplotype. After analysis of traits TBIN, TBINA, and TCHRONA, the results provided an indication on the status of haplotypes being favorable, intermediate, or unfavorable. In a second run, for each allele, the status of each individual was defined based on whether the haplotype allele occurred twice (homozygous), once (heterozygous), or not at all (all other combinations). Only haplotype alleles appearing in at least 10 animals were included in further analysis. The impact of each haplotype allele was estimated applying the SAS procedure GLIMMIX under the following threshold model:

$$PR\left(Y_{ijkl}=1\right)=\varphi \left(\mu +herd_{i}+parity_{j}+DIM_{k}+haplotype_{l}\right),$$ (2)

where *PR* is the probability of occurence, *Y*_*ijkl*_ is the binary trait (TBIN, TBINA, and TCHRONA; 1 = affected and 0 = unaffected), *φ* is the probit link function, *μ* is the overall mean, *herd*_*i*_ is the fixed effect of the farm (*i* = 1, …, 7), *parity*_*j*_ is the fixed effect for parity (primipar or multipar), *DIM*_*k*_ is the fixed effect for the lactation stage grouped into classes (*k* = 1, …, 8 for DIM <50, 50–99, 100–149, 150–199, 200–249, 250–299, 300–349, and >350), and *haplotype*_*l*_ is the fixed effect for haplotype (l = 0, 1, and 2 for none, heterozygous, and homozygous, respectively).

Haplotype alleles that would warrant further analysis were chosen on the basis of the *p*-values of significance as well as the occurrence of homozygous and heterozygous individuals, the number of homozygous individuals (N ≥ 5), and a clear differentiation between LSM estimates of homozygous and heterozygous animals.

For position 90,100,118 (HapMap60356-rs29024194, BTA11), three haplotypes were identified with one being unfavorable in the homozygous status and two being favorable. For each of the three haplotypes, sires being heterozygous were identified with one sire exhibiting haplotype 669/701 (both haplotypes favorable), another sire showing 701/164 haplotypes (favorable/unfavorable), and a further sire with 669/164 haplotypes (favorable/unfavorable). For all three sires, two homozygous daughters for each haplotype were selected for sequencing of the target gene. For position 44,597,888 (rs41603040—BTA-45551-no-rs, BTA19), two haplotypes were identified with one being favorable and one being unfavorable in the homozygous status. For five heterozygous sires, a total of 11 homozygous daughters were sequenced for the target gene.

### Fluorescence Resonance Energy Transfer Analysis

High-throughput examination of rs208894039 (*CMPK2*) and rs109521151 (*ASB16*) was carried out using fluorescence resonance energy transfer (FRET) for 2,485 (*CMPK2*) and 2,471 (*ASB16*) animals from batch 1 with phenotype information regarding the established binary BDD traits.

Since in the genome database Ensembl (Genome assembly ARS-UCD1.2) (Zerbino et al., 2018), A and G are, respectively, specified as reference and alternative alleles for rs208894039, genotypes were denoted as homozygous reference (A/A), heterozygous variant (A/G), or homozygous variant (G/G). For SNP rs109521151, A is denoted as the reference allele and G as the variant allele (genome assembly ARS-UCD1.2). Genotype information was statistically tested against the traits using the SAS procedure GLIMMIX under a threshold model considering herd, parity, and days in milk (DIM) as fixed effects.

## Results

### Phenotype Data From Generally Affected (TBIN) and Chronic Proliferative (TCHRONA) Affected Cows Highlight Ubiquitous Occurrence of BDD in All Herds

Phenotype data were collected from cows in a total of 13 large dairy herds. Table 1 displays the results of descriptive statistics for the binary phenotypes as defined across multiple visits. TBIN = 0 denotes a healthy cow throughout visits, while TBIN is coded as 1 for any lesion detected, be it an infectious stage (M2) or a chronic case of M4. TBINA refers to infectious cases scored as 1, while all other stages are coded as 0. Finally, TCHRONA refers to scores given for signs of chronicity with proliferations, coded as 1, while all other cases are scored as 0.

**TABLE 1:** Overview of the data by herds and phenotypic trait variables.

Herd	N	TBIN		TBINA		TCHRONA	
		Mean	Std	Mean	Std	Mean	Std
1	528	0.57765	0.49440	0.10606	0.30821	0.37121	0.48359
2	230	0.83043	0.37607	0.49565	0.50107	0.40870	0.49267
3	860	0.59302	0.49156	0.05814	0.23414	0.33837	0.47343
4	548	0.45620	0.49853	0.03102	0.17354	0.23540	0.42464
5	176	0.55682	0.49818	0.14205	0.35009	0.25568	0.43749
6	97	0.41237	0.49482	0.15464	0.36344	0.12371	0.33096
7	81	0.60494	0.49191	0.09877	0.30021	0.18519	0.39087
8	461	0.44035	0.49697	0.00651	0.08049	0.41215	0.49276
9	301	0.31229	0.46420	0.00664	0.08138	0.27243	0.44595
10	549	0.76321	0.42550	0.05647	0.23103	0.37121	0.48359
11	166	0.86747	0.34009	0.17470	0.38086	0.40870	0.49267
12	503	0.31610	0.46542	0.02783	0.16466	0.33837	0.47343
13	540	0.61481	0.48709	0.27037	0.44456	0.23540	0.42464

Initially, seven herds were included in the study and phenotyped in 2015 and 2016, and these data are denoted as batch 1. In 2018, cows in further six herds were phenotyped forming batch 2 of the data. Herds included in batch 1 are numbered from one to seven, while second batch herds are numbered from 8 to 13. Differences between herds appear to be of high magnitude when considering infectious cases only as indicated by means for TBINA. Herds 3, 4, 10, and 12 exhibit rather low numbers of M2 lesions and in herds 8 and 9 infectious cases are even scarcer. In contrast, herds 2 and 13 show rather high values for infectious cases. Phenotypes, TBIN and TCHRONA, however, show that in all herds BDD is abundant. It should be noted that phenotypes TBIN and TCHRONA are closely related, since many M4 cases exhibit chronic signs, that is, proliferation and/or hyperkeratotic lesions.

### GWAS Shows Significant Association for BDD on Chromosomes 11 and 19

**FIGURE 1:**
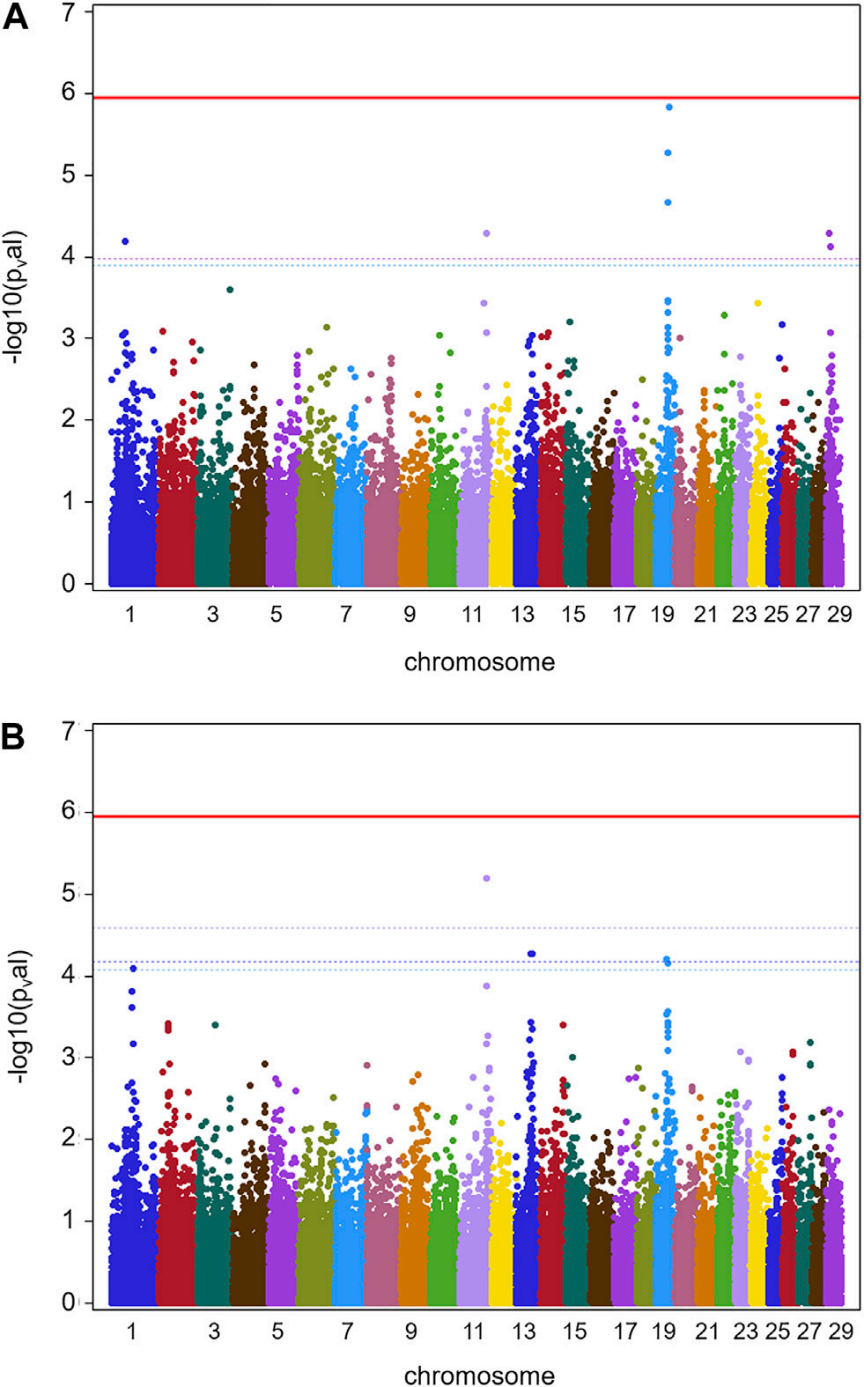
GWAS results for data batch 1 (herds 1–7). **(A)** Manhattan plot derived from the results of GWAS analysis (Ensembl genome assembly UMD3.1) for trait TBIN. **(B)** Manhattan plot obtained from the results of GWAS analysis for trait TCHRONA. In all plots -log10 *p*-values of detected SNP sorted by chromosomes are shown. Red line indicates genome-wide (5%) false discovery rate (FDR). Colored dotted lines indicate chromosome-wise (5%) FDR with line and corresponding chromosome of the same color.

**FIGURE 2:**
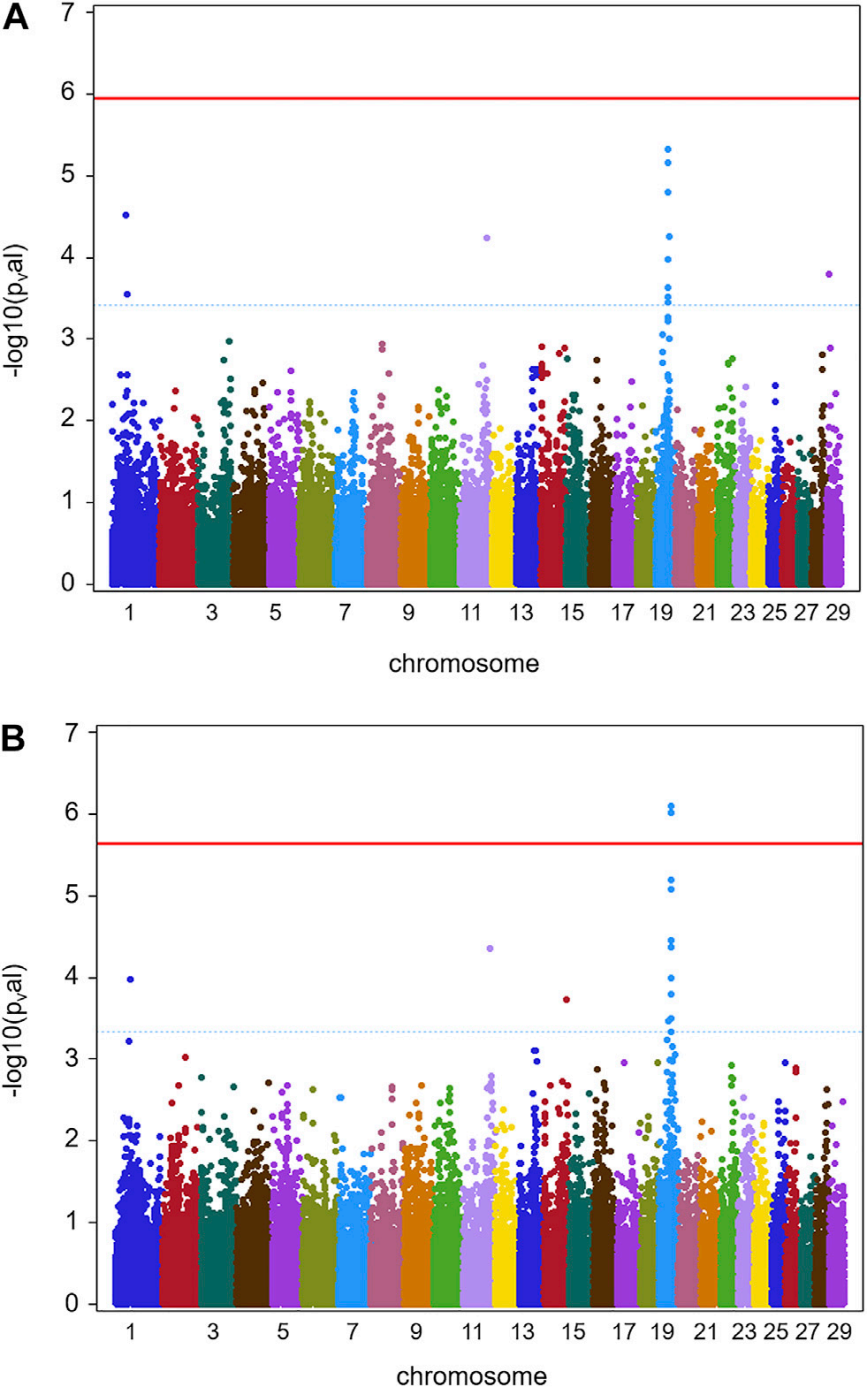
GWAS results for full data (batch 1 and 2) (herds 1–13). **(A)** Manhattan plot obtained from the results of GWAS analysis (Ensembl genome assembly UMD3.1) for trait TBIN. **(B)** Manhattan plot derived from the data of GWAS analysis for trait TCHRONA. In all plots -log10 *p*-values of detected SNP sorted by chromosomes are shown. Red line indicates genome-wide (5%) false discovery rate (FDR). Blue dotted line indicates chromosome-wise (5%) FDR with line and corresponding chromosome of the same color.

Results for GWAS are presented in Figure 1 (batch 1) and Figure 2 (batch 1 + 2). Details on the significance of individual SNP are given in Supplementary Tables S3A, S3B. Figure 1A indicates one clearly interesting chromosomal region for phenotype TBIN on BTA19, based on chromosome-wise FDR values for three SNPs. Another region of interest is located on BTA11 at position 90,100,118 with one SNP chromosome-wise significant FDR and trait TCHRONA focusing on chronic proliferative cases of BDD (Figure 1B). No significant *p*-values were found for any chromosomal region and phenotype TBINA (data not shown). Preliminary analyses (not shown) for this phenotype applying simpler approaches for GWAS neglecting co-variances between animals arising from genetic relationships, yielded *p*-values that differed among each other but provided no indications for any chromosomal region with a suspected effect on the trait. It has to be concluded that given the data shown in Table 1 with most herds exhibiting only low numbers of active M2 lesions, the definition of phenotype TBINA is not useful. For phenotype TCHRONA (Figure 1B), two SNPs with the chromosome-wise FDR value were also found on BTA19. For TBIN and TCHRONA, additional chromosomal regions of interest were found on BTA13 and BTA29. However, as these were not detectable in a consistent manner for both phenotypes, these regions were not considered for further investigation.

Figure 2 shows GWAS results for the full data of batches 1 and 2 combined for phenotypes TBIN and TCHRONA. Due to the low incidence of M2 cases in herds 3, 4, 8, 9, 10, and 12, again no significant estimates of SNP effects could be obtained for phenotype TBINA. Hence, the results for TBINA are not shown. For TBIN (Figure 2A), nine chromosomal regions exhibit a chromosome-wise significance using FDR values. On BTA11, a non-significant -log10 *p*-value of 4.23 is obtained for HAPMAP60356-rs29024194 at position 90,100,118. Other SNPs around this position show -log10 *p*-values between 2.0 and 3.0. For BTA19, a total of 11 SNPs show chromosome-wise significant FDR values; among them, two SNPs show a Bonferroni-corrected genome-wide significance for trait TCHRONA (Figure 2B). Considering phenotype TCHRONA (Figure 2B), the general picture for chromosomal regions of interest appears to be similar to TBIN.

### Selection of Potential Causative Candidate Genes for BDD Flanking HAPMAP60356-rs29024194 (BTA11) and BTA-45551-no-rs on BTA19

**FIGURE 3:**
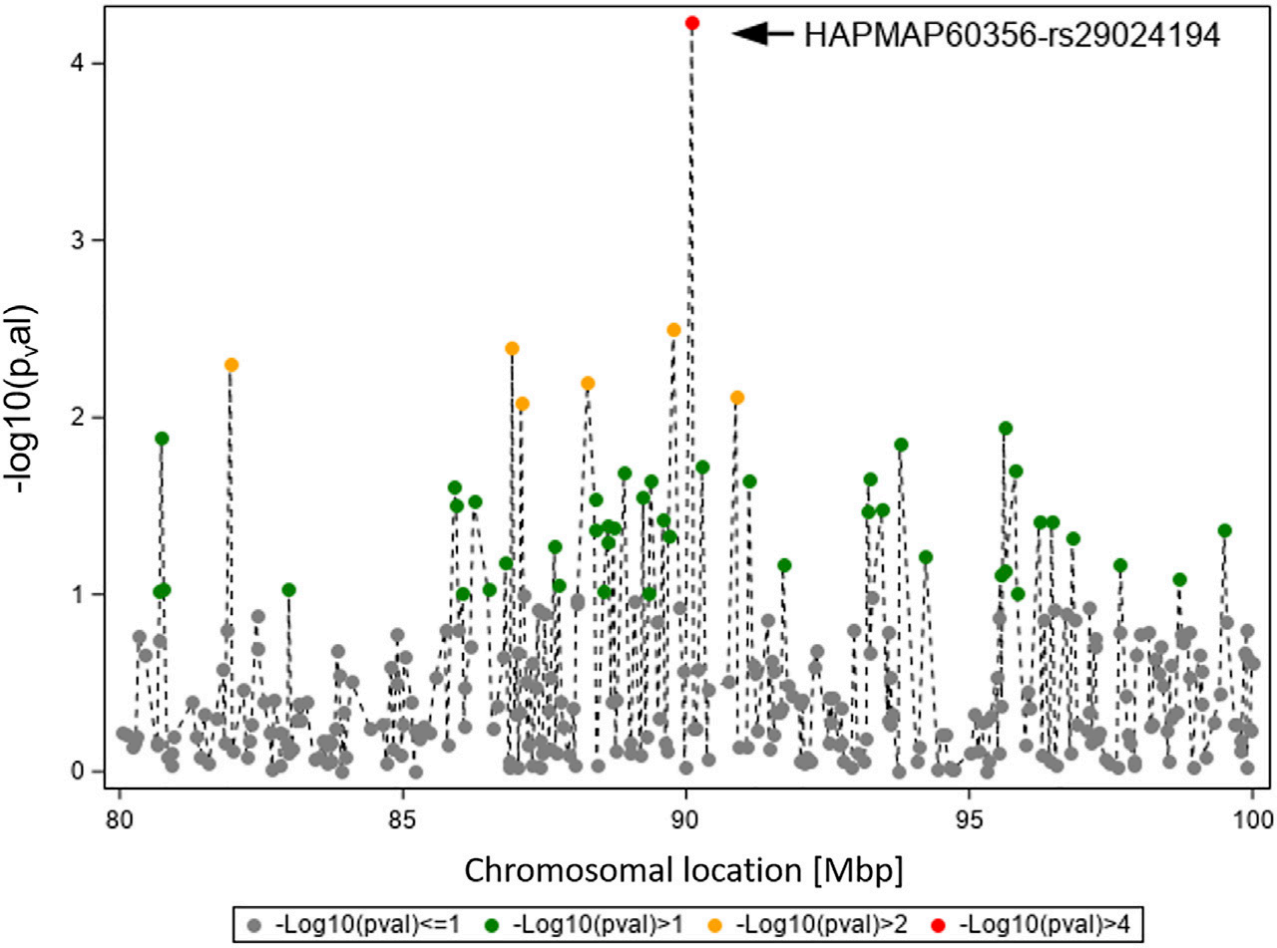
Overview of the chromosomal region around HAPMAP60356-rs29024194 on BTA11 for trait TBIN. The -log10 *p*-values of detected SNP are shown. The strongest effect could be calculated for SNP HAPMAP60356-rs29024194 (black arrowhead). In general, SNP with *p*-values < 1.5 and SNP with SNP-genotypes < 100 animals for the rarer homozygous genotype are not displayed.

Figure 3 displays the GWAS results (Ensembl genome assembly UMD3.1) for traits TBIN and BTA11, focusing on the region between 80 and 100 Mbp. The signal for HAPMAP60356-rs29024194 at position 90,100,118 is flanked by less pronounced signals. The closest gene to HAPMAP60356-rs29024194 is *CMPK2* (cytidine/uridine monophosphate kinase 2; Ensembl ID ENSBTAG00000019979) (Zerbino et al., 2018), which lies approximately 41.1 kb upstream of this polymorphism. Since studies in different species indicate an important role of *CMPK2* regarding immunomodulatory signaling pathways (Lee and O’Brien, 1995; Zhang et al., 2020), especially in association with bacterial infections (Zhong et al., 2018; Feng et al., 2021), this gene was selected as a candidate gene for a potential influence on BDD.

**FIGURE 4:**
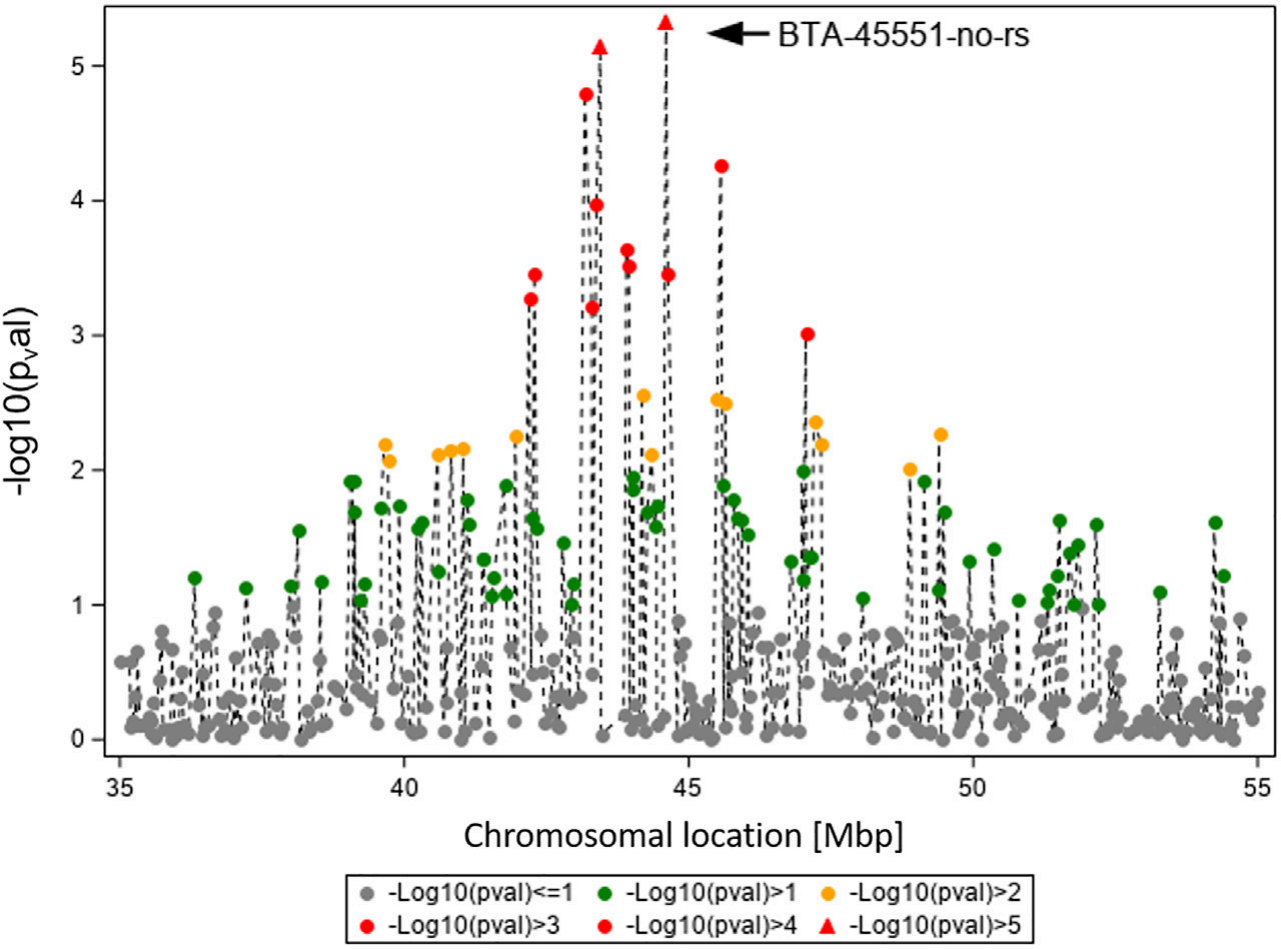
Overview of the chromosomal region around BTA-45551-no-rs on BTA19 for trait TBIN. The -log10 *p*-values of detected SNP are shown. The strongest effect could be calculated for SNP BTA-45551-no-rs (black arrowhead). Analogous to Figure 3, SNP with *p*-values < 1.5 and SNP with SNP-genotypes < 100 animals for the rarer homozygous genotype are not displayed.

In Figure 4, the GWAS results (Ensembl genome assembly UMD3.1) for TBIN and BTA19 are shown for the region between 35 and 55 Mbp. Of particular note is BTA-45551-no-rs at position 44,597,888, chromosome-wise significant for both traits and both datasets. From this SNP, the gene *ASB16* (ankyrin repeat and SOCS box containing 16; Ensembl ID ENSBTAG000019658) (Zerbino et al., 2018) is located 16.2 kb downstream. For ASB proteins, interaction with numerous effector molecules within inflammatory signaling pathways has already been shown (Andresen et al., 2014). In addition, ASB proteins play an important role in the regulation of protein activities through their function as the substrate recognition unit in a subset of E3 ubiquitin ligases, which is significant in maintaining a balanced immunological response (Anasa et al., 2018; Hou et al., 2021). Considering this, *ASB16* was selected as a potential BDD candidate gene.

### Haplotype and FRET Analyses Reveal Significant Influence of rs208894039 Downstream of *CMPK2* and rs109521151 in *ASB16* on BDD

The two chromosomal regions identified in the GWAS and the respective candidate genes were subsequently used as a basis for a haplotype analysis and subsequent identification of animals for sequencing of the candidate genes. This analysis revealed a remarkable difference between samples at genomic position 90,083,819 (BTA11), which lies 307 bp downstream of *CMPK2*. This distinct position with rs208894039 is not included on the Illumina Bovine BeadChip used in this study. Sequencing of *ASB16* was performed analogously and uncovered a clear variation between samples at genomic position 43,995,946 (BTA19) within exon 5. This position has dbSNP number rs109521151. The alternative G allele of this SNP causes a missense variation leading to incorporation of arginine instead of histidine at amino acid position 447 of ASB16 protein.

Genotypes for rs208894039 and rs109521151 were the results from a FRET analysis of batch 1, with a total of N = 2,485 of evaluable genotypes among the 2,520 total number of samples. Table 2 summarizes the results for the threshold model analysis of rs208894039 (A/G, BTA11) downstream of *CMPK2* at position 90,083,819 that was identified after sequencing of this genetic region. Apart from the genotype, as fixed effects in the model herd, parity, and classes for the lactation stage were included. For TBIN, substantial differences exist between LSMeans (LSM) of the three genotypes. WT/WT animals show lowest values for the status = 1 of being diseased while MT/MT animals show highest values. Heterozygous animals appear to react similar to MT/MT animals, thus pointing to an incomplete dominance of the G allele for the disease status. Differences comparing MT/MT with WT/WT and WT/WT with Het are highly significant (*p* < 0.0001). Comparisons of MT/MT against Het were not significant. For TBINA, including only M2 lesions for status = 1 (diseased), none of the comparisons for the three genotypes were significant. In the bottom part of Table 2, the results for TCHRONA are displayed, thus focusing on chronic proliferative cases vs. other animals taken as healthy. The results shown are highly analogous to the comparisons for TBIN although less pronounced. In summary, for the polymorphism rs208894039, substantial differences between genotypes can be identified despite the lower frequency of the MT allele (*q* = 0.24). More than half of the animals possess the advantageous genotype WT/WT. However, only one copy of the MT allele drastically changes susceptibility to BDD suggesting a dominant effect of the alternative allele over the reference allele.

**TABLE 2:** FRET analysis (Ensembl genome assembly ARS-UCD1.2) results of rs208894039 (BTA11). LSMEANs for the probability of being diseased according to trait definitions TBIN, TBINA, and TCHRONA are given along with the number of animals in each sub-cell and the statistical contrasts between genotypes.

Trait	Genotype	No. of cows by status		Total	LSMean	s.e	*p*-value of contrasts	
		0	1				MT/MT -	WT/WT -
TBIN	MT^c^/MT	48	93	141	0.699	0.040	—	0.0001
	Het^b^	347	568	915	0.638	0.020	0.16	<0.0001
	WT^a^/WT	670	749	1429	0.527	0.018	—	—
	Total	1065	1420	2485	—	—		
TBINA	MT/MT	120	21	141	0.164	0.037	—	0.072
	Het	813	102	915	0.109	0.013	0.102	0.825
	WT/WT	1271	158	1429	0.106	0.011	—	—
	Total	2204	281	2485	—	—		
TCHRONA	MT/MT	88	53	141	0.336	0.042	—	0.0008
	Het	586	329	915	0.300	0.020	0.3952	<0.0001
	WT/WT	1038	391	1429	0.210	0.015	—	—
	Total	1712	773	2485	—	—		

a WT/WT: homozygous for the reference allele.

b Het: heterozygous.

c MT/MT: homozygous for the alternative allele.

Table 3 displays the results for rs109521151 (A/G, BTA19) at position 43,995,946. For this polymorphism within *ASB16*, N = 2,471 animals had evaluable genotypes. For phenotype TBIN, drastic differences can be observed between MT/MT and WT/WT genotypes, with WT/WT genotypes being advantageous. MT/MT animals are less frequent, with an allele frequency of p (MT) = 0.128. Heterozygous animals exhibit a diseases status of almost intermediate incidence when compared to MT/MT and WT/WT animals. Contrast of MT/MT vs. WT/WT animals was significant with *p* = 0.0011. Comparing WT/WT animals to heterozygous animals yielded significant differences with *p* < 0.0001. Contrasts between MT/MT and heterozygous animals were not significant. The results for phenotype TBINA follow the same pattern as shown for TBIN; however, all comparisons between genotypes were not significant. For TCHRONA, that is, when contrasting chronic proliferative cases vs. all other animals, elevated values for the status of phenotype equal to 1 can be found for MT/MT animals, while WT/WT animals show substantially lower values and heterozygous animals have intermediate values. Contrasts between MT/MT and WT/WT animals as well as WT/WT and heterozygous animals were highly significant (*p* < 0.001), while contrasts between MT/MT and heterozygous animals were significant at a level of *p* = 0.03. Again, for phenotype TCHRONA, a similar pattern was observed in comparison to phenotype TBIN. In summary of the results in Tables 2, 3, in both cases, the alleles associated with increased susceptibility for BDD are substantially less frequent.

**TABLE 3:** FRET analysis (Ensembl genome assembly ARS-UCD1.2) results of rs109521151 (BTA19). Analogous to Table 2, the LSMEANs for the probability of being diseased according to trait definitions TBIN, TBINA, and TCHRONA are given along with the number of animals in each sub-cell and the statistical contrasts between genotypes.

Trait	Genotype	No. of cows by status		Total	LSMean	s.e	*p*-value of contrasts	
		0	1				MT/MT -	WT/WT -
TBIN	MT^c^/MT	13	44	57	0.778	0.056	—	0.0011
	Het^b^	176	344	520	0.679	0.023	0.1369	<0.0001
	WT^a^/WT	866	1028	1894	0.545	0.017	—	—
	Total	1055	1416	2471	—	—		
TBINA	MT/MT	47	10	57	0.145	0.048	—	0.3368
	Het	439	81	520	0.133	0.018	0.7955	0.0895
	WT/WT	1704	190	1894	0.104	0.010	—	—
	Total	2190	281	2471	—	—		
TCHRONA	MT/MT	28	29	57	0.448	0.069	—	0.0005
	Het	330	190	520	0.304	0.023	0.0328	0.0008
	WT/WT	1344	550	1894	0.232	0.015	—	—
	Total	1702	769	2471	—	—		

a WT/WT: homozygous for the reference allele.

b Het: heterozygous.

c MT/MT: homozygous for the alternative allele.

**FIGURE 5:**
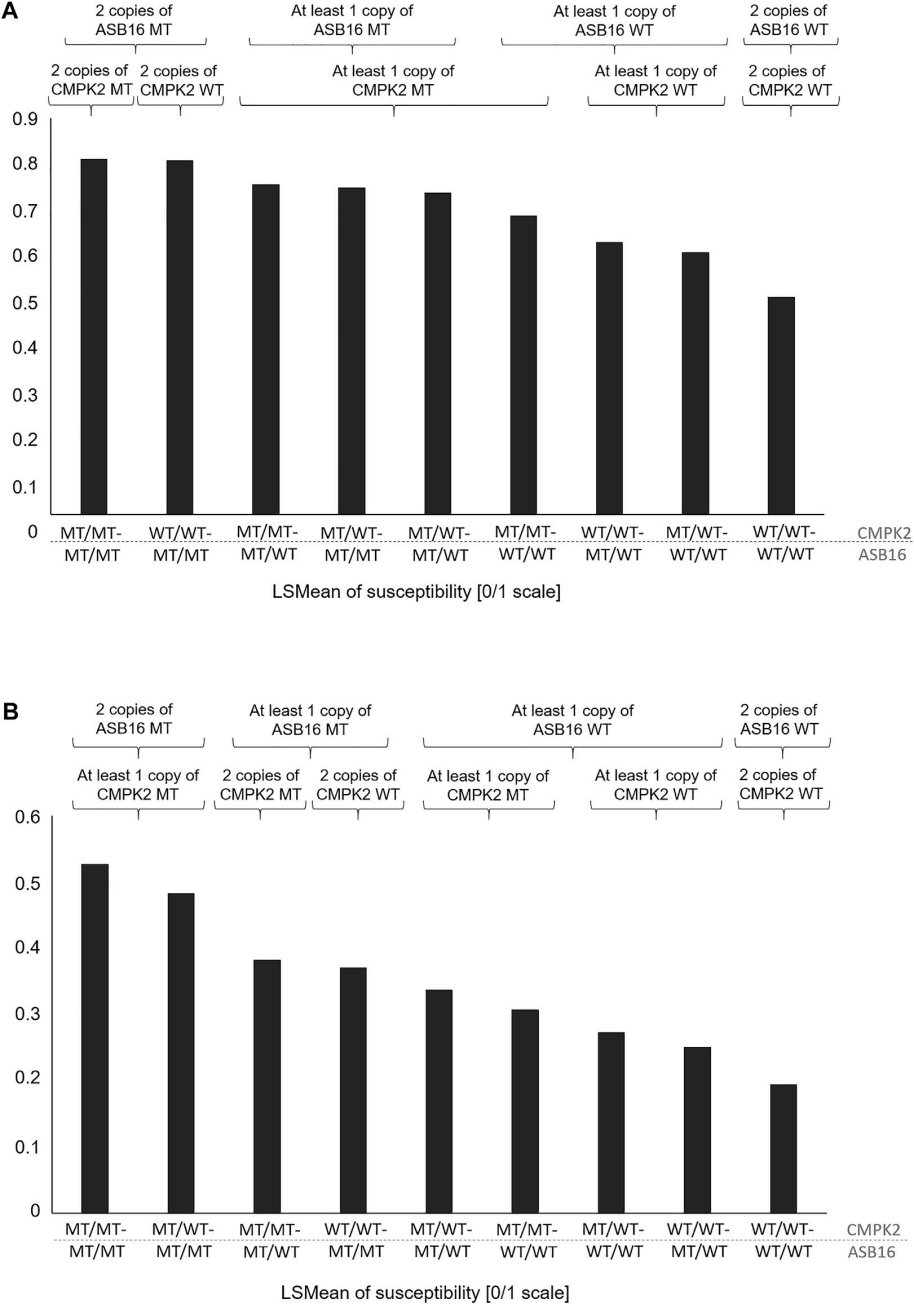
LSMeans of the disease status for combinations of genotypes rs208894039 (*CMPK2*) (first part of the genotype name) and rs109521151 (*ASB16*) (second part of the name). **(A)** LSMeans ranked by the value from the highest to lowest for phenotype TBIN. **(B)** LSMeans ranked by the value from the highest to lowest for phenotype TCHRONA. WT/WT: homozygous for the reference allele; MT/MT: homozygous for the alternative allele.

In Table 4, the combination of analysis of the SNP rs208894039 and rs109521151 is shown. A total of N = 2,461 animals had evaluable genotypes for a combination of both polymorphisms. Genotypes are denoted as such that the BTA11 polymorphism is given first, followed by the BTA19 genotype. For interpretation of Table 4, it has to be taken into consideration that the desirable and more common allele for both the *CMPK2* variant and the *ASB16* variant is WT (see Tables 2, 3). This is confirmed as shown in Table 4 as the WT/WT—WT/WT genotype shows lowest values for TBIN and TCHRONA for the status of being diseased. The combined WT/WT—WT/WT genotype is the most common of all combinations, thus underpinning possible effects of a positive indirect selection. In contrast, the MT/MT—MT/MT genotype exhibits most undesirable values for all phenotypes. For all combinations of combined genotypes, contrasts were estimated and tested for their significance. In the case of phenotype TBINA, not a single contrast approached significance, thus emphasizing again that scoring of the M2 stage animals vs. all other animals does not appear to reveal genomic effects. Hence, the results for TBINA may be dropped from further interpretation. For phenotypes, TBIN and TCHRONA, many LSMeans as well as many contrasts showed significance (Supplementary Table S4) and thus indeed warrant a detailed presentation for the disease status. In Figure 5, the LSMeans for both phenotypes, TBIN (Figure 5A) and TCHRONA (Figure 5B), were ordered from the highest to the lowest value, and the resulting bars were grouped according to the number of copies of the two variants. In both cases, rs109521151 (*ASB16*) is shown to have a dominant effect and rs208894039 (*CMPK2*) a modulatory effect.

**TABLE 4:** Combination of FRET results for BTA11 (rs208894039) x BTA19 (rs109521151) genotypes for traits TBIN, TBINA, and TCHRONA. The BTA11 variant is listed first, followed by the BTA19 genotype. Significant contrasts between genotypes are given in Supplementary Table S4.

Combination of genotype	No. of cows by status		Total	TBIN		TBINA		TCHRONA	
	0	1		LSMean	s.e	LSMean	s.e	LSMean	s.e
WT^a^/WT–MT^c^/MT	4	19	23	0.804	0.0883	0.086	0.05349	0.3706	0.1012
WT/WT–Het^b^	102	157	259	0.618	0.0332	0.127	0.02300	0.2507	0.0284
WT/WT–WT/WT	557	578	1135	0.495	0.0201	0.102	0.01206	0.1944	0.0153
Het—MT/MT	7	19	16	0.743	0.0869	0.179	0.08152	0.4832	0.1026
Het–Het	62	158	220	0.731	0.0321	0.127	0.02497	0.3370	0.0346
Het–WT/WT	275	384	659	0.595	0.0230	0.099	0.01425	0.2731	0.0211
MT/MT–MT/MT	2	6	8	0.808	0.1324	0.248	0.17120	0.5274	0.1925
MT/MT–Het	11	28	39	0.750	0.0691	0.205	0.01788	0.3817	0.0808
MT/MT–WT/WT	33	59	92	0.679	0.0507	0.141	0.04345	0.3065	0.0497
Total	1053	1408	2461						

a WT/WT: homozygous for the reference allele.

b Het: heterozygous.

c MT/MT: homozygous for the alternative allele.

## Discussion

Genome-wide association studies using SNP data and appropriate phenotype information have been successfully applied to identify candidate genes and further to detect causal variants for complex traits in cattle (Pausch et al., 2014; Swalve et al., 2014). For our study, we conducted precise phenotyping and applied distinct binary definitions of BDD traits to explicitly distinguish between generally affected (TBIN), actively affected (TBINA), and chronically (proliferation of skin) affected (TCHRONA) animals.

For complex traits, individual genomic loci can only explain a fraction of the entire genetic variance with influence on the development and progress of disease (Hayes and Goddard, 2001; van der Spek et al., 2015). For the case of BDD in dairy cattle, in our study, this hypothesis was confirmed by the identification of two SNPs on two different chromosomes. Polymorphisms identified were significantly linked to the general BDD status (TBIN) and chronic proliferation of the skin (TCHRONA). Further research in this regard may help clarify the dynamics of BDD within a herd, as chronic lesions are considered reservoirs for *Treponema* spp. making herds susceptible to endemic BDD (Gomez et al., 2014). The trait definition of TBINA is obviously not sensitive enough as animals coded with 0 contain a mix of healthy and chronical cases. Animal selection for initial sequencing of the candidate loci was facilitated by using information on phased haplotypes. Although several BDD candidate genes involving the regulation of the cell cycle, immune system, and skin barrier have been described (Scholey et al., 2012; Refaai et al., 2013; Scholey et al., 2013; El-Shafaey et al., 2017), no functional mutation has been directly linked to BDD to the authors’ knowledge. We would like to propose *CMPK2* and *ASB16* as novel candidate genes for BDD due to the detected variants rs208894039 and rs109521151 exhibiting significant association with disease susceptibility and probability of chronic progression.

For *CMPK2*, very few cattle-specific publications are available so far although indications of a role in inflammation-associated signaling pathways do exist (Blomström et al., 2015; Nilson, 2016). Moreover, since *CMPK2* is conserved in higher vertebrates (Chen et al., 2008), information from orthologs of other species is likely to be valid. *CMPK2* expression could be induced by substances like LPS (lipopolysaccharide), a potent bacterial inflammatory agent, and also by IFN (interferon) (Feng et al., 2021; Kim et al., 2021). IFN is secreted by immune cells upon pathogen recognition or reacting to other cytokines and is required for the regulation of the immune response. Thus, IFN also contributes to the activation of pro-inflammatory macrophages, which are involved in attacking pathogens by means of phagocytosis (Martinez and Gordon, 2014). *CMPK2* expression has also been widely used for monitoring to trace inflammation upon bacterial infection with Lyme disease spirochete *Borrelia burgdorferi* in mice (Ma et al., 2014) and human (Salazar et al., 2009). BDD in cattle is regarded as a polymicrobial disease, with treponemes, which also belong to the spirochete family, playing a major role in pathogenesis (Zinicola et al., 2015). In our study, we have demonstrated that rs208894039 (11:g.90083819 A > G, ARS-UCD1.2) has significant influence on BDD traits (Table 2) in dairy cattle. Compared to the homozygous reference (A/A), heterozygous (A/G) and homozygous alternative (G/G) animals are significantly more likely to be affected by BDD in general (TBIN) and, if affected, to develop chronic (proliferative) lesions (TCHRONA). As no significant differences exist between the variant and the heterozygous condition, one G allele is obviously sufficient to account for an increased susceptibility to be affected in general as well as for developing chronic stages. The polymorphism rs208894039 is located in the 3’ downstream region of *CMPK2* (Zerbino et al., 2018). Sequences located downstream of a gene can influence its expression because proper transcriptional regulation of genes in terms of site, timing, and level of expression may require coordinated action of multiple cis-regulatory elements. Control elements defined as enhancers or repressors may be located over long distances both upstream and downstream of a gene and affect its expression (Wittkopp and Kalay, 2012; Smith and Shilatifard, 2014). Sequence variants in such elements may affect adequate gene expression and thus represent a potential cause of genetic diseases in addition to defects directly within the gene sequence (Kleinjan and van Heyningen, 2005; Heinz et al., 2013). Different studies demonstrate effects on gene expression and binding behavior of transcription factors to the enhancer in the presence of a SNP within a regulatory element (Tough and Prinjha, 2017; Peng et al., 2020). In the downstream of the human *CMPK2* gene, several enhancer sequences have already been characterized (Zerbino et al., 2018), suggesting a possible regulation of gene expression. Currently, no such elements have been clearly characterized the downstream of bovine *CMPK2*, but a similar constellation as in the human system would be possible. Furthermore, the expression of *CMPK2* may be influenced by transcription factors because database research [TFBIND (Tsunoda and Takagi, 1999), PROMO (Messeguer et al., 2002), and ConTra v3 (Kreft et al., 2017)] revealed binding sites for pro-inflammatory associated factors such as AP-1 (activator protein 1), NFkB (nuclear factor kappa B), STAT1 (signal transducer and activator of transcription 1), and IRF1 (interferon regulatory factor 1) in the promoter region*.* In addition to the role of mitochondrial CMPK2 in nucleotide synthesis, KEGG pathway analysis shows that the activity of CMPK2 also contributes to production of amino acids and to DNA repair mechanisms, maintaining the vitality of activated pro-inflammatory macrophages (Blaser et al., 2016; Kanehisa et al., 2018). In a study using a *Cmpk2* knockout mouse model, it was shown that correct regulation of *CMPK2* activity is important to prevent an uncontrolled inflammatory reaction due to the activation of the NLRP3 (NLR family pyrin domain containing 3) inflammasome complex, which leads to the production of the pro-inflammatory cytokine IL-1β (interleukin 1β) in activated macrophages (Zhong et al., 2018). This function of CMPK2 was also recently confirmed in a study using fish as a model organism, suggesting a cross-species function (Feng et al., 2021). CMPK2 appears to be involved in maintaining cellular functions such as phagocytosis and cytokine secretion in activated macrophages. Hence, it seems conceivable that variant-related differences in *CMPK2* activity could contribute to differences in disease affinity and progression.

ASB16 is one of the 18 mammalian members of the conserved ankyrin repeat and the SOCS (suppressor of cytokine signaling) box family (Kohroki et al., 2005). Members of the SOCS box protein family are implicated in the assembly of a subtype of E3 ubiquitin ligase complexes (Kohroki et al., 2005). Consequently, cellular processes such as proliferation, metabolism, and immune response can be regulated *via* proteasome-mediated degradation of specific substrate proteins (Yoshimura et al., 2007; Trengove and Ward, 2013; Kazi et al., 2014). So far, only a few specific substrates of ASB proteins are known. Identified potential substrates of ASB16 like HIF1AN (hypoxia inducible factor 1 subunit alpha inhibitor), FTH1 (ferritin heavy chain 1), and HSPA1 (heat shock protein family A (Hsp70) member 1A) (Andresen et al., 2014) can be involved in inflammatory signaling pathways in immune cells and in migration behavior of keratinocytes. Regulation of the own activity of ASB16 also appears to be probable (Andresen et al., 2014). The confirmed expression of ASB16 in human immune cells and also skin tissue (EMBL-EBI expression Atlas, release 04/2018, Cambridgeshire, United Kingdom) suggests a regulatory function for inflammatory processes in response to infections. Due to the highly conserved structure of the SOCS box, conclusions on possible binding partners or substrates of the ASB16 protein in cattle are conceivable (Kamura et al., 1998; Anasa et al., 2018). The bovine ASB16 protein comprises 453 amino acids, and the online protein domain prediction tool SMART (Letunic et al., 2006) localizes the SOCS box from amino acid 408–447. Variation at rs109521151 (19:g.43995946A>G, ARS-UCD1.2) entails a missense mutation, resulting in substitution of the last amino acid residue of this SOCS box (i.e., histidine > arginin; ENSBTAP00000026197.4:p.His447Arg). In general, substitution of a single amino acid can have a dramatic impact on activity and functionality of a given protein (Stefl et al., 2013). For example, sequence variations in the ankyrin repeat region and the SOCS box of ASB10, among others, alter protein stability and cellular localization and could be associated with glaucoma development (Pasutto et al., 2012; Keller and Wirtz, 2017). In addition, database research (PROMO (Messeguer et al., 2002), TFBIND (Tsunoda and Takagi, 1999), and ConTra v3 (Kreft et al., 2017)) revealed the presence of potential binding sites for factors like NFkB, AP-1, and STAT1 in the promoter region of bovine *ASB16*, suggesting activation in the context of immunological processes. The SNP rs109521151 seems to account for the observed differences regarding TBIN and TCHRONA with wild-type animals (A/A) overall better coping with disease. Database search (PredictSNP (Bendl et al., 2014) and Ensembl genome assembly UMD3.1 (Zerbino et al., 2018)) for the prediction of the SNP effect and the resulting amino acid exchange on the principle formation and function of the ASB protein revealed that the sequence variant can be classified as distinct tolerable rather than deleterious (Bendl et al., 2014; Zerbino et al., 2018). Yet, alterations such as in protein stability or the ability to interact with potential binding partners are conceivable. Likely, the amino acid exchange within the SOCS box of ASB16 affects the ubiquitin ligase activity, which might be contributing to an altered regulation of inflammatory processes in skin tissue and observed higher susceptibility to BDD.

Even though the exact role of the two genes in the regulation of inflammatory processes is still largely unclear, it may be hypothesized that the advantageous genotype combination of *ASB16* wild-type and *CMPK2* wild-type causes a favorable immune response, which is reflected in the lower susceptibility to the disease (Table 4; Figure 5). Since the vast majority of the analyzed cows (N = 1,135) had the favored genotype combination and only eight individuals had the most disadvantageous combination, it can be hypothesized that previous indirect effects of genetic selection have occurred leading to the enrichment of the advantageous combination. Between the most advantageous and the most disadvantageous combination, all other possible combinations are in a certain order with regard to disease susceptibility. When *ASB16* is present in the mutant variant, it is always associated with a trend toward high disease susceptibility for all traits, regardless of which genotype is present with respect to *CMPK2* (Figures 5A,B). The genetic configuration of *ASB16* therefore appears to have a greater impact on susceptibility to BDD than the variant status of *CMPK2*. As a part of an ubiquitin ligase complex, the ASB16 protein is involved in the degradation of a large number of target proteins and thus may be involved in the regulation of a broad spectrum of cellular pathways (Andresen et al., 2014). Studies on CMPK2, in contrast, suggest a more specific role in the functional integrity of pro-inflammatory macrophages (Xu et al., 2008; Zhong et al., 2018). In perspective, further studies should investigate whether there are direct interactions between the occurring genetic variants, as it is known that CMPK2 can also be regulated by ubiquitination, among others (Zhang et al., 2020), or whether the genes rather act in different signaling cascades that may, however, overlap in the context of inflammatory signaling networks.

To our knowledge, our study appears to be the largest GWAS on the genomic background of BDD susceptibility according to the number of animals with phenotypes and genotypes and at the same time considering repeated observations. A trained team focusing solely on BDD with the team leader being present at all farm visits collected all observations. The results from GWAS for BDD in dairy cattle are limited in total and hampered by either small numbers, and/or not accounting for the etiology by using repeated observations, or imprecise phenotypes. However, some analogies on the identification of the chromosomal regions as shown in this study do exist. Thus, among many other regions, chromosomal regions on BTA11 at 90 Mbp and on BTA19 at 30 Mbp and further positions have already been identified to influence BDD (Naderi et al., 2018; Croué et al., 2019; Lai et al., 2020). A study with phenotype definitions very similar to our analysis identified genes apparently involved in the etiology of BDD. Among them were *LASP1* on BTA19 and *DAB2IP* on BTA11 (Kopke et al., 2020), two genes that are close to the chromosomal regions we identified, which further supports our results.

In conclusion, this study represents one step further to determine genetic predispositions influencing the pathogenesis of BDD. As recently has been shown, genetic selection can aid substantially in the eradication of infectious diseases in livestock (Hulst et al., 2021). Our results could therefore contribute to classical genomic selection approaches and/or to the development of a BDD prescreening test, which now actually comes into reach. Such a test could find applications in cattle breeding by scanning for susceptible animals and excluding them from further propagation. The management of BDD-prone individuals could consequently be adapted, for example, by extended prevention programs. Deeper insights into potentially altered inflammatory processes due to existing polymorphisms related to *CMPK2* and *ASB16* could contribute to the use of more animal-specific immunomodulatory therapies in the future. This would improve animal welfare and allow farmers to be proactive and prevent reservoirs of infection in their farms instead of only reacting to advanced lesions. Nonetheless, since the etiology of BDD is considered multifactorial, genetic screening will just be one part of an integrated prevention programs. The abolishment of unsanitary conditions along with an improved claw and health management on farms are indispensable.

## Data Availability

The datasets presented in this study can be found in online repositories. The name of the repository/repositories and accession number(s) can be found at: https://osf.io/c243w/.

## References

[B1] Aguilar I. Misztal I. Legarra A. Tsuruta S. (2011). Efficient Computation of the Genomic Relationship Matrix and Other Matrices Used in Single-Step Evaluation. J. Anim. Breed. Genet. 128, 422–428. 10.1111/j.1439-0388.2010.00912.x 22059575

[B2] Anasa V. V. Ravanan P. Talwar P. (2018). Multifaceted Roles of ASB Proteins and its Pathological Significance. Front. Biol. 13, 376–388. 10.1007/s11515-018-1506-2

[B3] Andresen C. A. Smedegaard S. Sylvestersen K. B. Svensson C. Iglesias-Gato D. Cazzamali G. (2014). Protein Interaction Screening for the Ankyrin Repeats and Suppressor of Cytokine Signaling (SOCS) Box (ASB) Family Identify Asb11 as a Novel Endoplasmic Reticulum Resident Ubiquitin Ligase. J. Biol. Chem. 289, 2043–2054. 10.1074/jbc.M113.534602 24337577 PMC3900953

[B4] Bendl J. Stourac J. Salanda O. Pavelka A. Wieben E. D. Zendulka J. (2014). PredictSNP: Robust and Accurate Consensus Classifier for Prediction of Disease-Related Mutations. PLoS Comput. Biol. 10, e1003440. 10.1371/journal.pcbi.1003440 24453961 PMC3894168

[B5] Benjamini Y. Hochberg Y. (1995). Controlling the False Discovery Rate: A Practical and Powerful Approach to Multiple Testing. J. R. Stat. Soc. Ser. B Methodol. 57, 289–300. 10.1111/j.2517-6161.1995.tb02031.x

[B6] Berry S. L. Read D. H. Famula T. R. Mongini A. Döpfer D. (2012). Long-Term Observations on the Dynamics of Bovine Digital Dermatitis Lesions on a California Dairy After Topical Treatment with Lincomycin HCl. Veterinary J. 193, 654–658. 10.1016/j.tvjl.2012.06.048 22892182

[B7] Biemans F. de Jong M. C. M. Bijma P. (2019). A Genome-Wide Association Study for Susceptibility and Infectivity of Holstein Friesian Dairy Cattle to Digital Dermatitis. J. Dairy Sci. 102, 6248–6262. 10.3168/jds.2018-15876 31103307

[B8] Blaser H. Dostert C. Mak T. W. Brenner D. (2016). TNF and ROS Crosstalk in Inflammation. Trends Cell. Biol. 26, 249–261. 10.1016/j.tcb.2015.12.002 26791157

[B9] Blomström A.-L. Gu Q. Barry G. Wilkie G. Skelton J. K. Baird M. (2015). Transcriptome Analysis Reveals the Host Response to Schmallenberg Virus in Bovine Cells and Antagonistic Effects of the NSs Protein. BMC Genomics 16, 324. 10.1186/s12864-015-1538-9 25896169 PMC4404599

[B10] Cheli R. Mortellaro C. (1974). La dermatite digitale del bovino. Int. Meet. Dis. Cattle 8, 208–213.

[B11] Chen Y.-L. Lin D.-W. Chang Z.-F. (2008). Identification of a Putative Human Mitochondrial Thymidine Monophosphate Kinase Associated with Monocytic/Macrophage Terminal Differentiation. Genes. Cells 13, 679–689. 10.1111/j.1365-2443.2008.01197.x 18498354

[B12] Croué I. Michenet A. Leclerc H. Ducrocq V. (2019). Genomic Analysis of Claw Lesions in Holstein Cows: Opportunities for Genomic Selection, Quantitative Trait Locus Detection, and Gene Identification. J. Dairy Sci. 102, 6306–6318. 10.3168/jds.2018-15979 31056323

[B13] Döpfer D. Holzhauer M. Boven M. v. (2012). The Dynamics of Digital Dermatitis in Populations of Dairy Cattle: Model-Based Estimates of Transition Rates and Implications for Control. Veterinary J. 193, 648–653. 10.1016/j.tvjl.2012.06.047 22878094

[B14] Döpfer D. ter Huurne A. A. H. M. Cornelisse J. L. van Asten A. J. A. M. Koopmans A. Meijer F. A. (1997). Histological and Bacteriological Evaluation of Digital Dermatitis in Cattle, with Special Reference to Spirochaetes and Campylobacter Faecalis. Veterinary Rec. 140, 620–623. 10.1136/vr.140.24.620 9228692

[B15] El-Shafaey E.-S. Ateya A. Ramadan H. Saleh R. Elseady Y. Abo El Fadl E. (2017). Single Nucleotide Polymorphisms in IL8 and TLR4 Genes as Candidates for Digital Dermatitis Resistance/Susceptibility in Holstein Cattle. Anim. Biotechnol. 28, 131–137. 10.1080/10495398.2016.1242489 27813832

[B16] Evans N. J. Brown J. M. Scholey R. Murray R. D. Birtles R. J. Hart C. A. (2014). Differential Inflammatory Responses of Bovine Foot Skin Fibroblasts and Keratinocytes to Digital Dermatitis Treponemes. Veterinary Immunol. Immunopathol. 161, 12–20. 10.1016/j.vetimm.2014.05.005 25022220

[B17] Feng C. Tang Y. Liu X. Zhou Z. (2021). CMPK2 of Triploid Crucian Carp Is Involved in Immune Defense Against Bacterial Infection. Dev. Comp. Immunol. 116, 103924. 10.1016/j.dci.2020.103924 33186560

[B18] G K. K A. M K. L B. Hh S. Fb L. (2020). The Identification of Gene Ontologies and Candidate Genes for Digital Dermatitis in Beef Cattle from a Genome-Wide Association Study. Int. J. Vet. Sci. Res. 6, 027–037. 10.17352/ijvsr.000050

[B19] Gomez A. Anklam K. S. Cook N. B. Rieman J. Dunbar K. A. Cooley K. E. (2014). Immune Response Against Treponema Spp. And ELISA Detection of Digital Dermatitis. J. Dairy Sci. 97, 4864–4875. 10.3168/jds.2013-7616 24931522

[B20] Hayes B. Goddard M. E. (2001). The Distribution of the Effects of Genes Affecting Quantitative Traits in Livestock. Genet. Sel. Evol. 33, 209. 10.1186/1297-9686-33-3-209 11403745 PMC2705405

[B21] Heinz S. Romanoski C. E. Benner C. Allison K. A. Kaikkonen M. U. Orozco L. D. (2013). Effect of Natural Genetic Variation on Enhancer Selection and Function. Nature 503, 487–492. 10.1038/nature12615 24121437 PMC3994126

[B22] Holzhauer M. Bartels C. J. M. Döpfer D. van Schaik G. (2008). Clinical Course of Digital Dermatitis Lesions in an Endemically Infected Herd Without Preventive Herd Strategies. Veterinary J. 177, 222–230. 10.1016/j.tvjl.2007.05.004 17618149

[B23] Hou P. Jia P. Yang K. Li Z. Tian T. Lin Y. (2021). An Unconventional Role of an ASB Family Protein in NF-Κb Activation and Inflammatory Response During Microbial Infection and Colitis. Proc. Natl. Acad. Sci. U.S.A. 118. 10.1073/pnas.2015416118 PMC7826385 33431678

[B24] Hulst A. D. de Jong M. C. M. Bijma P. (2021). Why Genetic Selection to Reduce the Prevalence of Infectious Diseases Is Way More Promising Than Currently Believed. Genetics 217. 10.1093/genetics/iyab024 PMC8049556 33734349

[B25] Kamura T. Sato S. Haque D. Liu L. Kaelin W. G. Conaway R. C. (1998). The Elongin BC Complex Interacts with the Conserved SOCS-Box Motif Present in Members of the SOCS, Ras, WD-40 Repeat, and Ankyrin Repeat Families. Genes. Dev. 12, 3872–3881. 10.1101/gad.12.24.3872 9869640 PMC317264

[B26] Kanehisa M. Sato Y. Furumichi M. Morishima K. Tanabe M. (2018). New Approach for Understanding Genome Variations in KEGG. Nucleic Acids Res. 47, D590–D595. 10.1093/nar/gky962 PMC6324070 30321428

[B27] Kazi J. U. Kabir N. N. Flores-Morales A. Rönnstrand L. (2014). SOCS Proteins in Regulation of Receptor Tyrosine Kinase Signaling. Cell. Mol. Life Sci. 71, 3297–3310. 10.1007/s00018-014-1619-y 24705897 PMC11113172

[B28] Keller K. E. Wirtz M. K. (2017). Working Your SOCS Off: The Role of ASB10 and Protein Degradation Pathways in Glaucoma. Exp. Eye Res. 158, 154–160. 10.1016/j.exer.2016.06.003 27296073 PMC5149453

[B29] Kim H. Subbannayya Y. Humphries F. Skejsol A. Pinto S. M. Giambelluca M. (2021). UMP-CMP Kinase 2 Gene Expression in Macrophages Is Dependent on the IRF3-IFNAR Signaling axis. PLoS ONE 16, e0258989. 10.1371/journal.pone.0258989 34705862 PMC8550426

[B30] Kleinjan D. A. van Heyningen V. (2005). Long-Range Control of Gene Expression: Emerging Mechanisms and Disruption in Disease. Am. J. Hum. Genet. 76, 8–32. 10.1086/426833 15549674 PMC1196435

[B31] Kohroki J. Nishiyama T. Nakamura T. Masuho Y. (2005). ASB Proteins Interact with Cullin5 and Rbx2 to Form E3 Ubiquitin Ligase Complexes. FEBS Lett. 579, 6796–6802. 10.1016/j.febslet.2005.11.016 16325183

[B32] Kreft Ł. Soete A. Hulpiau P. Botzki A. Saeys Y. De Bleser P. (2017). ConTra V3: A Tool to Identify Transcription Factor Binding Sites Across Species, Update 2017. Nucleic Acids Res. 45, W490–W494. 10.1093/nar/gkx376 28472390 PMC5570180

[B33] Lai E. Danner A. L. Famula T. R. Oberbauer A. M. (2020). Genome-Wide Association Studies Reveal Susceptibility Loci for Digital Dermatitis in Holstein Cattle. Animals 10, 2009. 10.3390/ani10112009 PMC7693332 33142934

[B34] Laven R. A. Logue D. N. (2006). Treatment Strategies for Digital Dermatitis for the UK. Veterinary J. 171, 79–88. 10.1016/j.tvjl.2004.08.009 16427584

[B35] Lee C. G. O'Brien W. E. (1995). A Unique Member of the Thymidylate Kinase Family that Is Induced During Macrophage Activation. J. Immunol. 154, 6094–6102. 7751651

[B36] Letunic I. Copley R. R. Pils B. Pinkert S. Schultz J. Bork P. (2006). SMART 5: Domains in the Context of Genomes and Networks. Nucleic Acids Res. 34, D257–D260. 10.1093/nar/gkj079 16381859 PMC1347442

[B37] Ma Y. Bramwell K. K. C. Lochhead R. B. Paquette J. K. Zachary J. F. Weis J. H. (2014). Borrelia BurgdorferiArthritis-Associated LocusBbaa1 Regulates Lyme Arthritis and K/B×N Serum Transfer Arthritis Through Intrinsic Control of Type I IFN Production. J. I. 193, 6050–6060. 10.4049/jimmunol.1401746 PMC4258437 25378596

[B38] Martinez F. O. Gordon S. (2014). The M1 and M2 Paradigm of Macrophage Activation: Time for Reassessment. F1000Prime Rep. 6, 13. 10.12703/P6-13 24669294 PMC3944738

[B39] Messeguer X. Escudero R. Farre D. Nunez O. Martinez J. Alba M. M. (2002). PROMO: Detection of Known Transcription Regulatory Elements Using Species-Tailored Searches. Bioinformatics 18, 333–334. 10.1093/bioinformatics/18.2.333 11847087

[B40] Misztal I. Tsuruta S. Strabel T. Auvray B. Lee D. H. (2002). “BLUPF90 and Related Programs (BGF90), in: CD-ROM Communication,” in Proceedings of the 7th World Congress on Genetics Applied to Livestock Production, Montpellier, France, August 19-23, 2002.

[B41] Naderi S. Bohlouli M. Yin T. König S. (2018). Genomic Breeding Values, SNP Effects and Gene Identification for Disease Traits in Cow Training Sets. Anim. Genet. 49, 178–192. 10.1111/age.12661 29624705

[B42] Nilson S. (2016). “Comparative Analyses of Transcriptome Data from Beef Cattle Persistently Infected with Bovine Viral Diarrhea Virus,” (Nebraska, USA: University of Nebraska-Lincoln). Dissertation.

[B43] Orsel K. Plummer P. Shearer J. De Buck J. Carter S. D. Guatteo R. (2018). Missing Pieces of the Puzzle to Effectively Control Digital Dermatitis. Transbound. Emerg. Dis. 65 (Suppl. 1), 186–198. 10.1111/tbed.12729 29124910

[B44] Pasutto F. Keller K. E. Weisschuh N. Sticht H. Samples J. R. Yang Y.-F. (2012). Variants in ASB10 Are Associated with Open-Angle Glaucoma. Hum. Mol. Genet. 21, 1336–1349. 10.1093/hmg/ddr572 22156576 PMC3284122

[B45] Pausch H. Kölle S. Wurmser C. Schwarzenbacher H. Emmerling R. Jansen S. (2014). A Nonsense Mutation in TMEM95 Encoding a Nondescript Transmembrane Protein Causes Idiopathic Male Subfertility in Cattle. PLoS Genet. 10, e1004044. 10.1371/journal.pgen.1004044 24391514 PMC3879157

[B46] Peng T. Zhong L. Gao J. Wan Z. Fu W.-P. Sun C. (2020). Identification of Rs11615992 as a Novel Regulatory SNP for Human P2RX7 by Allele-Specific Expression. Mol. Genet. Genomics 295, 23–30. 10.1007/s00438-019-01598-0 31410611

[B47] Refaai W. Ducatelle R. Geldhof P. Mihi B. El-shair M. Opsomer G. (2013). Digital Dermatitis in Cattle Is Associated with an Excessive Innate Immune Response Triggered by the Keratinocytes. BMC Vet. Res. 9, 193. 10.1186/1746-6148-9-193 24090086 PMC3851557

[B48] Salazar J. C. Duhnam-Ems S. La Vake C. Cruz A. R. Moore M. W. Caimano M. J. (2009). Activation of Human Monocytes by Live Borrelia Burgdorferi Generates TLR2-Dependent and -Independent Responses Which Include Induction of IFN-β. PLoS Pathog. 5, e1000444. 10.1371/journal.ppat.1000444 19461888 PMC2679197

[B49] Sánchez-Molano E. Bay V. Smith R. F. Oikonomou G. Banos G. (2019). Quantitative Trait Loci Mapping for Lameness Associated Phenotypes in Holstein-Friesian Dairy Cattle. Front. Genet. 10, 926. 10.3389/fgene.2019.00926 31636655 PMC6787292

[B50] Sargolzaei M. Chesnais J. P. Schenkel F. S. (2014). A New Approach for Efficient Genotype Imputation Using Information from Relatives. BMC Genomics 15, 478. 10.1186/1471-2164-15-478 24935670 PMC4076979

[B51] Scholey R. A. Blowey R. W. Murray R. D. Smith R. F. Cameron J. Massey J. P. (2012). Investigating Host Genetic Factors in Bovine Digital Dermatitis. Veterinary Rec. 171, 624. 10.1136/vr.101251 23193036

[B52] Scholey R. A. Evans N. J. Blowey R. W. Massey J. P. Murray R. D. Smith R. F. (2013). Identifying Host Pathogenic Pathways in Bovine Digital Dermatitis by RNA-Seq Analysis. Veterinary J. 197, 699–706. 10.1016/j.tvjl.2013.03.008 23570776

[B53] Schöpke K. Gomez A. Dunbar K. A. Swalve H. H. Döpfer D. (2015). Investigating the Genetic Background of Bovine Digital Dermatitis Using Improved Definitions of Clinical Status. J. Dairy Sci. 98, 8164–8174. 10.3168/jds.2015-9485 26364113

[B54] Shearer J. K. Hernandez J. (2000). Efficacy of Two Modified Nonantibiotic Formulations (Victory) for Treatment of Papillomatous Digital Dermatitis in Dairy Cows. J. Dairy Sci. 83, 741–745. 10.3168/jds.S0022-0302(00)74936-8 10791790

[B55] Smith E. Shilatifard A. (2014). Enhancer Biology and Enhanceropathies. Nat. Struct. Mol. Biol. 21, 210–219. 10.1038/nsmb.2784 24599251

[B56] Speijers M. H. M. Baird L. G. Finney G. A. McBride J. Kilpatrick D. J. Logue D. N. (2010). Effectiveness of Different Footbath Solutions in the Treatment of Digital Dermatitis in Dairy Cows. J. Dairy Sci. 93, 5782–5791. 10.3168/jds.2010-3468 21094750

[B57] Stefl S. Nishi H. Petukh M. Panchenko A. R. Alexov E. (2013). Molecular Mechanisms of Disease-Causing Missense Mutations. J. Mol. Biol. 425, 3919–3936. 10.1016/j.jmb.2013.07.014 23871686 PMC3796015

[B58] Swalve H. H. Floren C. Wensch-Dorendorf M. Schöpke K. Pijl R. Wimmers K. (2014). A Study Based on Records Taken at Time of Hoof Trimming Reveals a Strong Association Between the IQ Motif-Containing GTPase-Activating Protein 1 (IQGAP1) Gene and Sole Hemorrhage in Holstein Cattle. J. Dairy Sci. 97, 507–519. 10.3168/jds.2013-6997 24237756

[B59] Tough D. F. Prinjha R. K. (2017). Immune Disease-Associated Variants in Gene Enhancers Point to BET Epigenetic Mechanisms for Therapeutic Intervention. Epigenomics 9, 573–584. 10.2217/epi-2016-0144 27925476

[B60] Tremblay M. Bennett T. Döpfer D. (2016). The DD Check App for Prevention and Control of Digital Dermatitis in Dairy Herds. Prev. Veterinary Med. 132, 1–13. 10.1016/j.prevetmed.2016.07.016 27664443

[B61] Trengove M. C. Ward A. C. (2013). SOCS Proteins in Development and Disease. Am. J. Clin. Exp. Immunol. 2, 1–29. 23885323 PMC3714205

[B62] Tsunoda T. Takagi T. (1999). Estimating Transcription Factor Bindability on DNA. Bioinformatics 15, 622–630. 10.1093/bioinformatics/15.7.622 10487870

[B63] Tuschil A. Lam C. Haslberger A. Lindley I. (1992). Interleukin-8 Stimulates Calcium Transients and Promotes Epidermal Cell Proliferation. J. Investigative Dermatology 99, 294–298. 10.1111/1523-1747.ep12616634 1512465

[B64] van der Spek D. van Arendonk J. A. M. Bovenhuis H. (2015). Genome-Wide Association Study for Claw Disorders and Trimming Status in Dairy Cattle. J. Dairy Sci. 98, 1286–1295. 10.3168/jds.2014-8302 25497826

[B65] Wittkopp P. J. Kalay G. (2012). Cis-Regulatory Elements: Molecular Mechanisms and Evolutionary Processes Underlying Divergence. Nat. Rev. Genet. 13, 59–69. 10.1038/nrg3095 22143240

[B66] Xu Y. Johansson M. Karlsson A. (2008). Human UMP-CMP Kinase 2, a Novel Nucleoside Monophosphate Kinase Localized in Mitochondria. J. Biol. Chem. 283, 1563–1571. 10.1074/jbc.M707997200 17999954

[B67] Yoshimura A. Naka T. Kubo M. (2007). SOCS Proteins, Cytokine Signalling and Immune Regulation. Nat. Rev. Immunol. 7, 454–465. 10.1038/nri2093 17525754

[B68] Zerbino D. R. Achuthan P. Akanni W. Amode M. R. Barrell D. Bhai J. (2018). Ensembl 2018. Nucleic Acids Res. 46, D754–D761. 10.1093/nar/gkx1098 29155950 PMC5753206

[B69] Zhang X. Zhang K. Zhang Y. (2020). Pigment Epithelium‑Derived Factor Facilitates NLRP3 Inflammasome Activation Through Downregulating Cytidine Monophosphate Kinase 2: A Potential Treatment Strategy for Missed Abortion. Int. J. Mol. Med. 45, 1436–1446. 10.3892/ijmm.2020.4517 32323732 PMC7138263

[B70] Zhong Z. Liang S. Sanchez-Lopez E. He F. Shalapour S. Lin X.-j. (2018). New Mitochondrial DNA Synthesis Enables NLRP3 Inflammasome Activation. Nature 560, 198–203. 10.1038/s41586-018-0372-z 30046112 PMC6329306

[B71] Zinicola M. Lima F. Lima S. Machado V. Gomez M. Döpfer D. (2015). Altered Microbiomes in Bovine Digital Dermatitis Lesions, and the Gut as a Pathogen Reservoir. PLoS ONE 10, e0120504. 10.1371/journal.pone.0120504 25781328 PMC4362943

