# Supplementary material for: Functional Variants Associated With CMPK2 and in ASB16 Influence Bovine Digital Dermatitis
Source: Front Genet. 2022 Jun 27;13:859595. doi: 10.3389/fgene.2022.859595 (PMC9271848; doi:10.3389/fgene.2022.859595)
Supplement: Supplementary file 1 [file DataSheet1.docx]

Supplementary Material


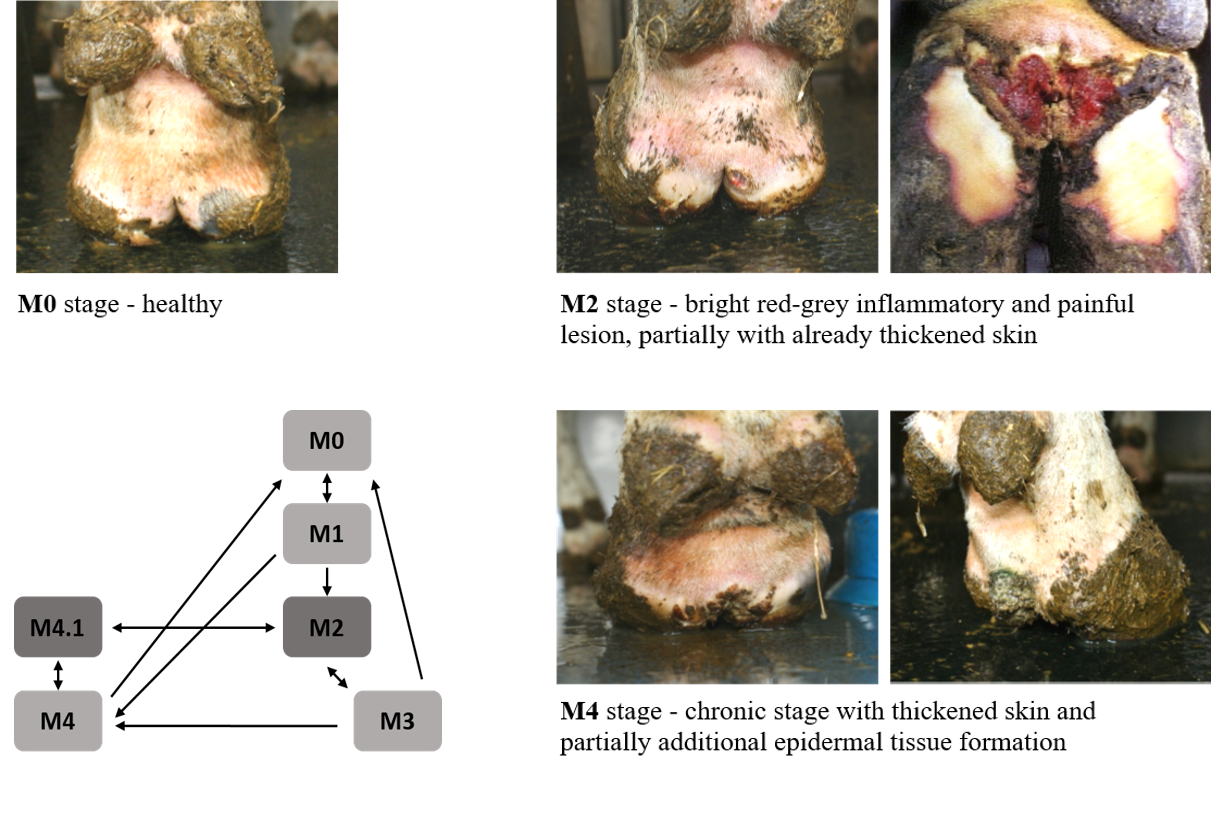


**Supplementary Figure 1:** M-scoring system for assessing the disease stage of DD

As shown in the scheme, the disease cycle can be divided into different stages (M0-M4). Stage M1 begins with local ischemia with discoloration and loss of epithelium and swelling as well as hyperemia around the lesion. Epithelial loss and damaged keratinocytes lead to an erosive and later proliferative stage (M2). After treatment, the lesion surface heals and becomes scab-like (M3). Chronic stages appear as hyperkeratotic or proliferative forms (M4), whereby new disease foci (M4.1) can also reappear (Döpfer et al., 1997; Döpfer et al., 2012).

**Supplementary Table 1:** *P*-values of F-test and predicted probabilities for each haplotype allele with at least five homozygous individuals for the traits TBIN, TBINA and TCHRONA and candidate gene region (*CMPK2*) on BTA11

| **HTID^a^** | **N HT^b^** | | **TBIN^c^** | | | | | **TBINA^c^** | | | | **TCHRONA^c^** | | | |
| --- | --- | --- | --- | --- | --- | --- | --- | --- | --- | --- | --- | --- | --- | --- | --- |
|  |  |  | ***p-*value^d^** | **probabilities (SE) (%)^e^** | | | ***p-*value^d^** | | **probabilities (SE) (%)^e^** | | | ***p-*value^d^** | **probabilities (SE) (%)^e^** | | |
|  | **1** | **2** |  | **0** | **1** | **2** |  |  | **0** | **1** | **2** |  | **0** | **1** | **2** |
| 14 | 515 | 23 | 1.28·10^-3^ | 56.18 (1.58) | 65.18 (2.36) | 64.69 (9.90) | 0.97 | | 11.84 (1.14) | 12.29 (1.77) | 8.10·10^-9^ (7.03·10^-5^) | 1.11·10^-3^ | 23.83 (1.40) | 31.76 (2.31) | 21.27 (8.40) |
| 17 | 354 | 10 | 0.88 | 58.31 (1.53) | 57.50 (2.88) | 65.09 (16.25) | 0.17 | | 12.01 (1.04) | 10.56 (1.87) | 33.21 (15.89) | 0.82 | 25.81 (1.41) | 24.25 (2.44) | 26.16 (13.35) |
| **164** | **510** | **43** | **1.04·10^-4^** | **55.72 (1.59)** | **66.25 (2.33)** | **64.95 (7.35)** | **0.02** | | **11.41 (1.05)** | **11.89 (1.73)** | **29.41 (7.99)** | **7.95·10^-4^** | **23.70 (1.39)** | **31.78 (2.34)** | **31.90 (7.35)** |
| 658 | 295 | 15 | 0.05 | 58.93 (1.51) | 54.38 (3.16) | 31.74 (12.09) | 0.98 | | 11.86 (1.11) | 12.36 (2.32) | 1.33·10^-7^ (1.39·10^-4^) | 0.46 | 25.51 (1.39) | 26.89 (2.79) | 14.11 (8.36) |
| **669** | **539** | **39** | **0.12** | **58.53 (1.55)** | **55.98 (2.46)** | **72.59 (7.42)** | **0.37** | | **11.70 (1.05)** | **11.71 (1.72)** | **20.57 (7.42)** | **0.04** | **26.61 (1.45)** | **22.38 (2.03)** | **15.94 (5.70)** |
| **701** | **774** | **87** | **2.73·10^-5^** | **61.57 (1.62)** | **52.82 (2.12)** | **45.59 (5.64)** | **0.55** | | **12.42 (1.15)** | **10.69 (1.38)** | **12.04 (3.89)** | **9.02·10^-3^** | **27.26 (1.54)** | **23.24 (1.79)** | **16.07 (3.88)** |
| 704 | 337 | 7 | 0.73 | 58.51 (1.51) | 56.16 (2.99) | 58.83 (19.03) | 0.31 | | 12.26 (1.13) | 9.16 (1.84) | 1.45·10^-6^ (1.25·10^-3^) | 0.50 | 25.24 (1.38) | 27.77 (2.63) | 36.24 (18.15) |

^a^Identifier for haplotype allele

^b^Number of individuals heterozygous (1) and homozygous (2) for haplotype allele

^c^Trait definitions as given in Materials and Methods.

^d^*p*-value of F-test evaluating the influence of genotype (haplotype allele)

^e^Predicted probabilities with standard error (SE) for individuals negative (0), heterozygous (1) or homozygous (2) for the haplotype allele. For calculation of the probabilities the inverse link function was applied to the LSM. Promising haplotype alleles are in bold type.

**Supplementary Table 2:** *P*-values of F-test and predicted probabilities for each haplotype allele with at least five homozygous individuals for the traits TBIN, TBINA and TCHRONA and candidate gene region (*ASB16*) on BTA19

| **HTID^a^** | **N HT^b^** | | **TBIN^c^** | | | | | **TBINA^c^** | | | | **TCHRONA^c^** | | | |
| --- | --- | --- | --- | --- | --- | --- | --- | --- | --- | --- | --- | --- | --- | --- | --- |
|  |  |  | ***p-*value^d^** | **probabilities (SE) (%)^e^** | | | ***p-*value^d^** | | **probabilities (SE) (%)^e^** | | | ***p-*value^d^** | **probabilities (SE) (%)^e^** | | |
|  | **1** | **2** |  | **0** | **1** | **2** |  |  | **0** | **1** | **2** |  | **0** | **1** | **2** |
| 9 | 336 | 10 | 0.69 | 0.5  (0.02) | 0.60  (0.03) | 0.47  (0.16) | 0.94 | | 0.12  (0.01) | 0.12  (0.02) | 0.10  (0.12) | 0.85 | 0.25  (0.01) | 0.26  (0.03) | 0.33  (0.15) |
| 12 | 455 | 23 | 0.70 | 0.59  (0.02) | 0.56  (0.03) | 0.62  (0.10) | 0.20 | | 0.12  (0.01) | 0.11  (0.02) | 0.25  (0.10) | 0.42 | 0.26  (0.01) | 0.23  (0.02) | 0.28  (0.09) |
| **52** | **525** | **49** | **<0.0001** | **0.54**  **(0.02)** | **0.68**  **(0.02)** | **0.83**  **(0.06)** | **0.02** | | **0.11**  **(0.01)** | **0.14**  **(0.02)** | **0.22**  **(0.06)** | **<0.0001** | **0.23**  **(0.01)** | **0.31**  **(0.02)** | **0.47**  **(0.07)** |
| 88 | 351 | 15 | 0.96 | 0.58  (0.02) | 0.58  (0.03) | 0.58  (0.13) | 0.98 | | 0.12  (0.01) | 0.12  (0.02) | 0.00  (0.00) | 0.20 | 0.25  (0.01) | 0.27  (0.03) | 0.09  (0.07) |
| **519** | **807** | **96** | **8.0 * 10^-4^** | **0.61**  **(0.02)** | **0.55**  **(0.02)** | **0.49**  **(0.05)** | **0.04** | | **0.14**  **(0.01)** | **0.10**  **(0.01)** | **0.08**  **(0.03)** | **0.005** | **0.27**  **(0.02)** | **0.24**  **(0.02)** | **0.16**  **(0.04)** |
| 521 | 443 | 20 | 0.59 | 0.58  (0.02) | 0.60  (0.03) | 0.59  (0.11) | 0.77 | | 0.12  (0.01) | 0.11  (0.02) | 0.10  (0.08) | 0.58 | 0.26  (0.01) | 0.24  (0.02) | 0.33  (0.10) |
| 524 | 515 | 34 | 0.33 | 0.59  (0.02) | 0.55  (0.02) | 0.55  (0.09) | 0.69 | | 0.12  (0.01) | 0.13  (0.02) | 0.12  (0.07) | 0.65 | 0.26  (0.01) | 0.25  (0.02) | 0.33  (0.08) |
| 780 | 329 | 9 | 0.27 | 0.59  (0.02) | 0.54  (0.03) | 0.64  (0.16) | 0.77 | | 0.12  (0.01) | 0.13  (0.02) | 0.00  (0.00) | 0.71 | 0.26  (0.01) | 0.25  (0.03) | 0.38  (0.16) |

^a^Identifier for haplotype allele

^b^Number of individuals heterozygous (1) and homozygous (2) for haplotype allele

^c^Trait definitions as given in Materials and Methods.

^d^*p*-value of F-test evaluating the influence of genotype (haplotype allele)

^e^Predicted probabilities with standard error (SE) for individuals negative (0), heterozygous (1) or homozygous (2) for the haplotype allele. For calculation of the probabilities the inverse link function was applied to the LSM. Promising haplotype alleles are in bold type.

**Supplementary Table 3A:** Significance of SNP by trait as given by position (UMD 3.1) for batch 1 and combined batch 1 and 2. Numbers indicate number of individuals per genotype when coded 0/1/2 for homogeneous/ heterogeneous/ homogeneous genotype. Numbers in red: SNP genome-wide significant after FDR correction, numbers in black: SNP significant under chromosome-wise FDR

|  |  |  | Batch 1 (N =2520) | | Batch 1+2 (N=5040) | |
| --- | --- | --- | --- | --- | --- | --- |
| chr | bp |  | TCHRONA | TBIN | TCHRONA | TBIN |
| 11 | 90100118 | all | 159/974/1387 |  |  |  |
|  |  | 0 | 95/627/1016 |  |  |  |
|  |  | 1 | 64/347/371 |  |  |  |
| 13 | 53684993 | all | 1241/1031/248 |  |  |  |
|  |  | 0 | 903/691/144 |  |  |  |
|  |  | 1 | 338/340/104 |  |  |  |
|  | 57367631 | all | 1620/777/123 |  |  |  |
|  |  | 0 | 1167/497/74 |  |  |  |
|  |  | 1 | 453/280/49 |  |  |  |
| 19 | 33533491 | all |  |  | 4449/573/18 |  |
|  |  | 0 |  |  | 2685/394/14 |  |
|  |  | 1 |  |  | 1764/179/4 |  |
|  | 39049389 | all | 433/1244/843 |  |  |  |
|  |  | 0 | 276/832/630 |  |  |  |
|  |  | 1 | 157/412/213 |  |  |  |
|  | 42193177 | all |  |  | 3418/1466/156 |  |
|  |  | 0 |  |  | 2198/822/73 |  |
|  |  | 1 |  |  | 1220/644/83 |  |
|  | 42289012 | all |  |  |  | 991/2462/1587 |
|  |  | 0 |  |  |  | 387/1070/789 |
|  |  | 1 |  |  |  | 604/1392/798 |
|  | 43170256 | all |  |  | 2782/1913/345 | 2782/1913/345 |
|  |  | 0 |  |  | 1626/1223/244 | 1140/915/191 |
|  |  | 1 |  |  | 1156/690/101 | 1642/998/154 |
|  | 43295532 | all |  |  | 3373/1497/170 |  |
|  |  | 0 |  |  | 2165/849/79 |  |
|  |  | 1 |  |  | 1208/648/91 |  |
|  | 43376032 | all |  | 1553/826/141 | 2960/1807/273 | 2960/1807/273 |
|  |  | 0 |  | 722/315/40 | 1907/1042/144 | 1415/742/89 |
|  |  | 1 |  | 831/511/101 | 1053/765/129 | 1545/1065/184 |
|  | 43441993 | all |  |  | 3145/1663/232 | 3145/1663/232 |
|  |  | 0 |  |  | 1864/1058/171 | 1314/793/139 |
|  |  | 1 |  |  | 1281/605/61 | 1831/870/93 |
|  | 43904899 | all |  |  | 1604/2485/951 | 1604/2485/951 |
|  |  | 0 |  |  | 1059/1499/535 | 798/1085/363 |
|  |  | 1 |  |  | 545/986/416 | 806/1400/588 |
|  | 43948803 | all |  |  | 1163/2488/1389 | 1163/2488/1389 |
|  |  | 0 |  |  | 791/1521/781 | 583/1119/544 |
|  |  | 1 |  |  | 372/967/608 | 580/1369/845 |
|  | 44345164 | all |  |  | 755/2361/1924 |  |
|  |  | 0 |  |  | 411/1421/1261 |  |
|  |  | 1 |  |  | 344/940/663 |  |
|  | 44597888 | all | 1924/543/53 | 1924/543/53 | 3736/1200/104 | 3736/1200/104 |
|  |  | 0 | 1370/345/23 | 882/186/9 | 2395/653/45 | 1778/443/25 |
|  |  | 1 | 554/198/30 | 1042/357/44 | 1341/547/59 | 1958/757/79 |
|  | 44632448 | all |  |  | 3147/1674/219 | 3147/1674/219 |
|  |  | 0 |  |  | 2024/960/109 | 1509/667/70 |
|  |  | 1 |  |  | 1123/714/110 | 1638/1007/149 |
|  | 45560806 | all |  | 1408/941/171 |  | 2717/1945/378 |
|  |  | 0 |  | 665/371/41 |  | 1321/799/126 |
|  |  | 1 |  | 743/570/130 |  | 1396/1146/252 |
| 29 | 13194072 | all |  | 130/954/1436 |  |  |
|  |  | 0 |  | 42/371/664 |  |  |
|  |  | 1 |  | 88/583/772 |  |  |
|  | 14618963 | all |  | 903/1188/429 |  |  |
|  |  | 0 |  | 335/524/218 |  |  |
|  |  | 1 |  | 568/664/211 |  |  |
|  |  |  |  |  |  |  |
|  | | |  |  |  |  |

**Supplementary Table 3B:** P-values of SNP by trait as given by position (UMD 3.1) for batch 1 and combined batch 1 and 2. Numbers in red: SNP genome-wide significant after FDR correction, numbers in black: SNP significant under chromosome-wide FDR

|  |  | Batch 1 (N =2520) | | Batch 1+2 (N=5040) | |
| --- | --- | --- | --- | --- | --- |
| chr.(#snp) | bp | TCHRONA | TBIN | TCHRONA | TBIN |
| 11  (1924) | 90100118 |  |  |  |  |
|  |  | 6,47379E-06 |  |  |  |
|  |  |  |  |  |  |
| 13  (1517) | 53684993 |  |  |  |  |
|  |  | 5,44149E-05 |  |  |  |
|  |  |  |  |  |  |
|  | 57367631 |  |  |  |  |
|  |  | 5,26097E-05 |  |  |  |
|  |  |  |  |  |  |
| 19  (1187) | 33533491 |  |  |  |  |
|  |  |  |  | 3,35916E-04 |  |
|  |  |  |  |  |  |
|  | 39049389 |  |  |  |  |
|  |  | 6,15348E-05 |  |  |  |
|  |  |  |  |  |  |
|  | 42193177 |  |  |  |  |
|  |  |  |  | 1,00283E-04 |  |
|  |  |  |  |  |  |
|  | 42289012 |  |  |  |  |
|  |  |  |  |  | 3,51583E-4 |
|  |  |  |  |  |  |
|  | 43170256 |  |  |  |  |
|  |  |  |  | 8,08217E-07 | 1,58488E-05 |
|  |  |  |  |  |  |
|  | 43295532 |  |  |  |  |
|  |  |  |  | 4,1969E-05 |  |
|  |  |  |  |  |  |
|  | 43376032 |  |  |  |  |
|  |  |  | 2,18093E-05 | 4,6272E-4 | 1,07374E-4 |
|  |  |  |  |  |  |
|  | 43441993 |  |  |  |  |
|  |  |  |  | 6,32309E-06 | 7,02477E-06 |
|  |  |  |  |  |  |
|  | 43904899 |  |  |  |  |
|  |  |  |  | 3,11459E-4 | 2,32973E-4 |
|  |  |  |  |  |  |
|  | 43948803 |  |  |  |  |
|  |  |  |  | 9,63901E-07 | 3,05593E-4 |
|  |  |  |  |  |  |
|  | 44345164 |  |  |  |  |
|  |  |  |  | 1,58639E-4 |  |
|  |  |  |  |  |  |
|  | 44597888 |  |  |  |  |
|  |  | 7,03608E-05 | 5,28392E-06 | 8,22312E-06 | 4,68135E-06 |
|  |  |  |  |  |  |
|  | 44632448 |  |  |  |  |
|  |  |  |  | 3,58329E-05 | 3,48568E-4 |
|  |  |  |  |  |  |
|  | 45560806 |  |  |  |  |
|  |  |  | 1,45974E-06 |  | 5,51654E-05 |
|  |  |  |  |  |  |
| 29  (936) | 13194072 |  |  |  |  |
|  |  |  | 5,21152E-05 |  |  |
|  |  |  |  |  |  |
|  | 14618963 |  |  |  |  |
|  |  |  | 7,52935E-05 |  |  |
|  |  |  |  |  |  |

**Supplementary Table 4:** List of significant contrasts between combinations of genotypes along with odds ratios for the three phenotypes TBIN, TBINA and TCHRONA. LSMEANS of individual genotypes are given in Table 4 and Figure 5

| Contrast  (Combination of 1st genotype –  combination of 2nd genotype)^d)^ | | TBIN | | TBINA | | TCHRONA | |
| --- | --- | --- | --- | --- | --- | --- | --- |
|  |  | p-value  of  contrast | Odds  Ratio^e)^ | p-value  of  contrast | Odds  Ratio^e)^ | p-value  of  contrast | Odds  Ratio^e)^ |
| c_MT^c)^/MT-x-a_WT^a)^/WT | c_WT/WT-x-a_WT/WT | 0.0011 | 2.161 |  |  | 0.0103 | 1.831 |
| c_MT/MT-x-a_MT/MT | c_WT/WT-x-a_WT/WT | 0.0892 | 4.301 |  |  | 0.0492 | 4.624 |
| c_MT/MT-x-a_Het^b)^ | c_WT/WT-x-a_WT/WT | 0.0028 | 3.065 | 0.0722 | 2.266 | 0.0071 | 2.558 |
| c_MT/MT-x-a_Het | c_Het-x-a_WT/WT | 0.0593 | 2.039 | 0.0678 | 2.334 |  |  |
| c_MT/MT-x-a_Het | c_WT/WT-x-a_Het |  |  |  |  | 0.0952 | 1.845 |
| c_WT/WT-x-a_WT/WT | c_WT/WT-x-a_MT/MT | 0.0109 | 0.239 |  |  | 0.0414 | 0.410 |
| c_WT/WT-x-a_WT/WT | c_WT/WT-x-a_Het | 0.0007 | 0.604 |  |  | 0.0356 | 0.721 |
| c_WT/WT-x-a_WT/WT | c_Het-x-a_WT/WT | <.0001 | 0.665 |  |  | <.0001 | 0.642 |
| c_WT/WT-x-a_WT/WT | c_Het-x-a_MT/MT | 0.0182 | 0.338 |  |  | 0.0011 | 0.258 |
| c_WT/WT-x-a_WT/WT | c_Het-x-a_Het | <.0001 | 0.360 |  |  | <.0001 | 0.475 |
| c_WT/WT-x-a_MT/MT | c_Het-x-a_WT/WT | 0.0701 | 2.780 |  |  |  |  |
| c_WT/WT-x-a_Het | c_Het-x-a_MT/MT |  |  |  |  | 0.0175 | 0.358 |
| c_WT/WT-x-a_Het | c_Het-x-a_Het | 0.0117 | 0.595 |  |  | 0.0342 | 0.658 |
| c_Het-x-a_WT/WT | c_Het-x-a_MT/MT |  |  |  |  | 0.0296 | 0.402 |
| c_Het-x-a_WT/WT | c_Het-x-a_Het | 0.0006 | 0.541 |  |  | 0.0688 | 0.739 |

^a)^ WT/WT: Homozygous for the reference allele; ^b)^ Het: Heterozygous; ^c)^ MT/MT: Homozygous for the alternative allele.

^d)^ c = SNP rs208894039 in CMPK2 on BTA11, a = SNP rs109521151 in ASB16 on BTA19

^e)^ Probability of 1^st^ genotype for being diseased in comparison to 2^nd^ genotype
